# *Flavescence Dorée* Strain-Specific Impact on Phenolic Metabolism Dynamics in Grapevine (*Vitis vinifera*) throughout the Development of Phytoplasma Infection

**DOI:** 10.1021/acs.jafc.3c06501

**Published:** 2023-12-19

**Authors:** Dino Davosir, Ivana Šola, Jutta Ludwig-Müller, Martina Šeruga Musić

**Affiliations:** † Department of Biology, Faculty of Science, University of Zagreb, Horvatovac 102a, 10000 Zagreb, Croatia; ‡ Faculty of Biology, Technische Universität Dresden, Zellescher Weg 20b, 01217 Dresden, Germany

**Keywords:** biotic stress, flavonoids, grapevine yellows, phenolic acids, polyphenols, plant−pathogen interactions, salicylic acid

## Abstract

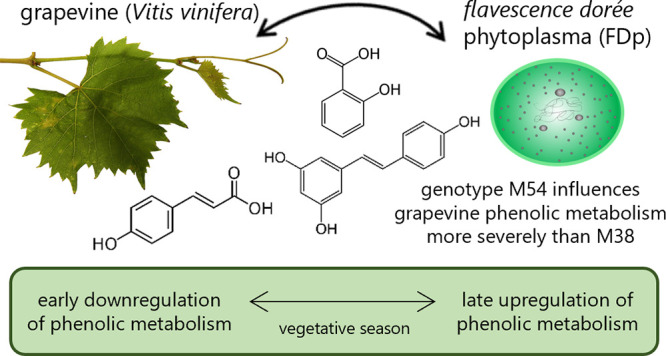

*Flavescence dorée* phytoplasma (FDp) is a phytopathogenic bacterium associated with Grapevine yellowS disease, which causes heavy damage to viticultural production. Epidemiological data revealed that some FDp strains appear to be more widespread and aggressive. However, there is no data on mechanisms underlying the variable pathogenicity among strains. In this research, we employed chromatographic and spectrophotometric techniques to assess how two strains of FDp influence the levels of grapevine phenolic compounds, which are frequently utilized as indicative markers of stress conditions. The results pointed to the upregulation of all branches of phenolic metabolism through the development of infection, correlating with the increase in antioxidative capacity. The more aggressive strain M54 induced stronger downregulation of phenolics’ accumulation at the beginning and higher upregulation by the end of the season than the less aggressive M38 strain. These findings reveal potential targets of FDp effectors and provide the first functional demonstration of variable pathogenicity between FDp strains, suggesting the need for future comparative genomic analyses of FDp strains as an important factor in exploring the management possibilities of FDp.

## 1 Introduction

Phytoplasmas (‘*Candidatus* Phytoplasma’) are a group of plant pathogenic bacteria that cause serious agricultural damage and affect plant food production worldwide. They are known to affect plants, as well as their insect vectors, in various ways, by manipulating their gene expression and signaling pathways.^1^ This causes severe reprogramming of plant transcriptome, proteome, and metabolome, which ultimately results in changes to morphology, growth, and normal physiological processes in plants, all in order to enable phytoplasma replication inside plant hosts and the attraction of vectors.^2^ These processes are mediated by phytoplasma effectors, which are abundant in phytoplasma genomes despite their severely reduced size. Some of those effectors are well characterized, but for most phytoplasmas, identification and functional analysis of their effectors are still lacking.^1^ High genome plasticity causes different strains of some phytoplasma species to have vastly different sets of effectors, as demonstrated for “*Ca.* P. solani” (*bois noir* phytoplasma, BNp).^3^ However, only a small number of studies attempted to examine the mechanisms of pathogenicity among different phytoplasma strains,^4^ despite the epidemiological data suggesting that some phytoplasma strains are known to be more pathogenic and widespread than others.^5,6^ In the case of grapevine *Flavescence dorée* phytoplasma (FDp; ribosomal group 16SrV), one of the causative agents of grapevine yellows disease, which impacts viticultural production across Europe, a high diversity of genotypes is recorded, based on a multilocus sequence typing scheme. Molecular epidemiology data identified genotypes that appear to be more aggressive. Although there is data on FDp epidemiology, it still lacks a formal description as a new phytoplasma species, with only one complete genome sequence published to date.^7^ Therefore, mechanisms of FDp pathogenicity in grapevine are still not well investigated, particularly on the aspect of differences in pathogenicity between FDp strains, neither on a molecular level nor on the impact on host physiology. One of the main obstacles in studying the mechanisms of plant–phytoplasma interactions is the inability to cultivate phytoplasmas in a pure culture *in vitro*, and infection assays are predominantly dependent on sampling the potentially infected vectors, which limits the success of such experiments.^8^ Therefore, most studies dealing with the investigation of the response of phytoplasma-infected plants still rely on the sampling of field-grown plants, which can also yield limited results due to the specificity of phytoplasma disease progression, as well as other factors.^9−11^

Plants possess several defense strategies against biotic stress and often produce specialized metabolites as a response to environmental factors, including pathogen attack. One of the main groups of specialized metabolites in plants are phenolic compounds, primarily produced through the phenylpropanoid pathway: phenolic acids, flavonoids, stilbenoids, and their polymers (condensed tannins, lignin, etc.).^12^ In some cases, it has been recorded that phytoplasma infection causes the upregulation of the general phenylpropanoid pathway, but in most cases particularly the flavonoid pathway.^2^ Even though substantial data suggest phytoplasmas modulate phenolic metabolism, no study so far has identified putative effectors that could mediate this process. Although the impact of BNp infection on the phenolic profile of grapevine has been previously thoroughly investigated,^13−15^ results for FDp are mostly lacking and only concentrated on a few groups of phenolic compounds.^9,11,16^ Therefore, considering the data that suggest that FDp affects grapevine phenolic metabolism, our aim was to assess if there is a strain-specific impact. Considering that phenolic metabolism is an often-used biomarker of various stress conditions in plants, our aim was to evaluate it as a potential indicator of variable pathogenicity levels among FDp strains. Of these phenolic compounds, salicylic acid (SA) is a key signaling molecule in the plant defense response against pathogens. When plants detect the presence of pathogens, including phytoplasmas, they often increase SA levels as part of their defense mechanism. Monitoring SA levels can therefore indicate the activation of the plant immune response.^17,18^ We compared the levels of groups of phenolic compounds (total phenolics, flavonoids, flavonols, anthocyanins, catechins, proanthocyanidins, phenolic acids, hydroxycinnamic acids, and tannins) and individual phenolic compounds between grapevine leaves infected with two different FDp strains (genotypes M38 and M54, based on the analysis of FDp methionine aminopeptidase gene sequences) and uninfected control through the development of infection, with the aim to analyze if the more predominant and aggressive genotype M54 would have a different impact than the less widespread and aggressive genotype M38.^5,6^

## 2 Materials and Methods

### 2.1 Sampling and Plant Material

Grapevine (*Vitis vinifera* L. var. ‘Pinot gris’) leaves from plants displaying symptoms of grapevine yellows (leaves yellowing and rolling, fruit drying) were collected from a vineyard in central continental Croatia (Sveti Ivan Žabno, Koprivnica-Križevci County), which was previously part of a multiyear surveillance for the presence of FD and BN phytoplasmas and a sampling location in previous studies.^5^ The vineyard was managed according to integrated pest and disease principles, with diseased plants removed if symptoms were observed each season. The leaves (three to four per replicate, three replicates per plant) were taken randomly from the same symptomatic (first year of symptom development) and asymptomatic plants at three time points during the development of infection: at the end of June, at the end of August, and at the end of September of 2021. Upon collection, the samples were stored in a field refrigerator, frozen under liquid nitrogen, and stored at −80 °C until freeze-drying. The plant material for metabolic analyses was freeze-dried using the Alpha 1–2 lyophilizer (Martin Christ Gefriertrocknungsanlagen GmbH, Osterode am Harz, Germany) at −55 °C and 0.05 mbar for approximately 32 h. Then, the material was ground to a fine powder in a mortar and pestle by using liquid nitrogen and stored for later analyses. Analyses for each of the tested groups were run in triplicate for each sample with three technical replicates.

### 2.2 Phytoplasma Detection and Identification

Total nucleic acids from grapevine midribs were extracted using a previously described CTAB-based method.^19^ Detection of FDp was performed using a triplex real-time PCR assay,^20^ utilizing a TaqMan Universal PCR Master Mix according to the manufacturer’s instructions and the reported primers and probes, on a 7300 Real-Time PCR System (Applied Biosystems, Waltham, USA), with PCR conditions as reported previously.^5^ Molecular typing of FDp-positive isolates was performed based on sequences of the methionine aminopeptidase (*map*) gene, which were amplified by nested PCR, using specific primers.^21^ Sequencing was performed by a commercial service (Genewiz-Azenta Life Sciences, Leipzig, Germany), and sequence editing, alignment, and phylogenetic analysis were performed as described previously.^22^

### 2.3 Analysis of Grapevine Phenolics Classes

The extraction of phenolic compounds from grapevine leaves was carried out with 70% (v/v) ethanol as a solvent.^23^ For extraction, a volume of 1 mL of 70% ethanol was mixed with 20 mg of the lyophilized powdered plant material. To improve the extraction of phenolics, the mixture of plant material and solvent was incubated using a digital tube rotator (Thermo Scientific, Shanghai, China) at 20 rpm for 60 min at room temperature. After the incubation, the extracts were centrifuged at 13,000*g* and 4 °C for 5 min. Afterward, the supernatant was transferred to a new tube and the samples were stored at −20 °C until further analyses.

The total phenolic content and content of groups of phenolic compounds (flavonoids, flavonols, anthocyanins, catechins, proanthocyanidins, phenolic acids, hydroxycinnamic acids, and tannins) were assessed by colorimetry-based methods, using UV/vis spectrophotometry. Detailed protocols for each method are given in Supporting Information 1.

### 2.4 RP-HPLC Analysis of Individual Phenolic Compounds

For qualitative and quantitative analyses of individual phenolic compounds from grapevine leaf ethanolic extracts, tentative analysis was carried out using reversed-phase high-performance liquid chromatography (RP-HPLC). To hydrolyze glycosylated phenolic compounds and produce aglycones for analysis, acidic hydrolysis was carried out with 1.2 M HCl for 2 h, at 80 °C and 300 rpm. The RP-HPLC analyses were performed using an Agilent (Santa Clara, USA) 1100 Series device equipped with a UV/vis detector. The separation was carried out on a Poroshell 120 SB-C_18_ nonpolar column (4.6 × 75 mm, 2.7 μm particle size) using the Zorbax Rx-C_18_ guard column (4.6 × 12.5 mm, 5 μm particle size), using a previously reported and validated method.^24^

The phenolic compounds were identified based on the comparison with the retention times and UV spectra of commercial standards. All standards of phenolic compounds were of HPLC grade and were obtained from Extrasynthese (Genay, France). For the quantitative analyses, external standards of identified compounds were used, and calibration curves were obtained by injecting known concentrations (0.25–0.01 mg/mL) of the mixed standard solutions. The quantitative analysis of myricetin was carried out at 254 nm, for epicatechin and cinnamic acid at 280 nm, for resveratrol, ferulic acid, and *p*-coumaric acid at 310 nm, and for quercetin and kaempferol at 360 nm. The results were expressed as either mg/g DW or μg/g DW. Representative chromatograms are presented in Supporting Information 2.

### 2.5 GC-MS Analysis of Salicylic Acid Content

The extraction of SA was performed as described,^25^ with some modifications. Extraction buffer (65% isopropanol:35% 200 mM imidazole, pH 7.0) was added to 100 mg of pulverized lyophilized tissue. Also, 1 μg of heavy-labeled SA-D_4_ (CDN Isotopes, Pointe-Claire, Canada) was added to each sample as an internal standard. The mixture was incubated for 1 h, at 4 °C in the dark on a rotary shaker. The samples were centrifuged, and the supernatant was taken to a new tube; 100 μL of deH_2_O was added. Isopropanol was removed under the flow of N_2_, after which 250 μL of deH_2_O was added. Then, pH of the aqueous phase was set to 3.0 using 2 M HCl. Then, 400 μL of ethyl acetate was added and the content was vortexed and centrifuged. The upper organic phase was taken into a glass vial, and the extraction procedure with ethyl acetate was repeated once more. The organic phase was dried under N_2_, after which 200 μL of methanol was added. For derivatization of SA to the more volatile methyl salicylate (MeSA), 200 μL of cold 20 mM trimethylsilyldiazomethane in diethyl ether was added. For methylation, samples were incubated for 30 min at RT, after which the content was dried under N_2_. Then, the dried content was dissolved in 50 μL of ethyl acetate for further analysis.

Gas chromatography coupled with mass spectrometry (GC-MS) analysis of SA content was carried out on a Varian Saturn 2100 ion-trap mass spectrometer using electron impact ionization at 70 eV, connected to a Varian CP-3900 gas chromatograph equipped with a CP-8400 autosampler (Varian, Walnut Creek, USA), with conditions as reported previously.^26^ MeSA was identified according to the retention time on GC compared with an authentic methylated standard (Sigma-Aldrich, Merck KGaA, Darmstadt, Germany), and the amount of SA was calculated using the isotope dilution equation with the ions at *m*/*z* 120 (endogenous SA) and *m*/*z* 124 (SA-D_4_) and expressed as ng/g DW.

### 2.6 Antioxidative Capacity Analyses

Analysis of the antioxidative capacity of grapevine leaves’ extracts was performed using three assays: ABTS (2,2′-azino-bis(3-ethylbenzothiazoline-6-sulfonic acid)) radical scavenging assay, DPPH (2,2-diphenyl-1-picrylhydrazyl) radical scavenging assay, and FRAP (ferric ion reducing antioxidant power) assay, as reported previously.^27^ Results were expressed as Trolox equivalent antioxidative capacity (TEAC), in milligrams of Trolox equivalents per g of DW (mg of TE/g of DW), based on calibration curves of standard antioxidant Trolox (6-hydroxy-2,5,7,8-tetramethylchroman-2-carboxylic acid, Sigma-Aldrich GmbH, Taufkirchen, Germany) solutions of known concentrations.

### 2.7 Statistical Analysis

Statistical analyses were conducted in Statistica 13.1 (StatSoft Inc., USA). The data were checked for distribution using the Shapiro–Wilk test and variance using Levene’s test before proceeding with the analysis. The analysis of statistically significant differences between the samples at each time point was carried out using one-way analysis of variance (ANOVA), followed by *post hoc* Duncan’s multiple range test. Statistically significant differences between the samples at *p* ≤ 0.05 were marked with different letters. To compare the dynamics of the parameters tested through the development of infection, values were normalized to the values of corresponding uninfected controls to eliminate the effect of seasonal dynamics on the tested parameters and the values from different time points for the same sample were compared. Pearson’s correlation coefficients (*r*) were calculated between the absolute and relative values of the tested parameters, with statistical significance at *p* ≤ 0.05. Principal component analysis (PCA) and hierarchical clustering analysis based on the Euclidian distance were performed to establish the relationship between samples based on the tested metabolic parameters.

## 3 Results and Discussion

Phytoplasma identification employing a real-time PCR assay revealed that grapevines displaying symptoms of GY were FDp-infected. Further sequencing and phylogenetic analysis of FDp *map* gene amplicons from infected grapevine revealed two different FDp genotypes among the infected plants, M54 and M38, both belonging to the mapFD2 genetic cluster. Genotype M54 was previously reported to be predominant in French vineyards,^6^ as well as in Italy,^28^ and an aggressive nature in both symptoms and spread of this genotype was reported in Croatia,^5^ whereas genotype M38 appears to be less aggressive and less dominant.^5,6^ Considering only one FDp genome sequence published so far, corresponding to the isolate with an M54 genotype,^7^ and obstacles that analyses of phytoplasma genomes face,^3^ detailed genomic and functional analyses to elucidate the differences in pathogenicity of FDp strains are as of yet unattainable. Therefore, in the scope of this study, we used grapevine phenolic metabolism as an indirect biomarker to assess the potential differences in pathogenicity between FDp strains corresponding to the genotypes M54 and M38 in grapevine compared to uninfected plants.

The upregulation of phenolic metabolism upon phytoplasma infection on transcriptomic and metabolomic levels has been previously reported.^2^ In our study, except at the first time point, total phenolics (TP) were higher in infected leaves at all time points (Table 1). This is in accordance with previous results of BNp impact of TP in grapevine,^15,29^ pointing to an increase in TP as a general response of grapevine leaves to phytoplasma infection. However, a suppression of phenolic metabolism at the first time point in both M54- and M38-infected leaves was not previously reported, probably due to these studies focusing more on the later stages of disease development, when more pronounced symptoms can be observed. This downregulation of phenolic biosynthesis could be an FDp strategy to limit the production of protective phenolic compounds early in the vegetative season, when the phytoplasma titer is still low. A similar strategy by which pathogens disrupt the phenolic biosynthesis were observed in case of *Mycosphaerella pinodes* infection.^30^ Future studies should focus on unraveling the exact mechanism of this process. Interestingly, TP was higher in M38-infected leaves at the second time point but at the third time point TP was higher in M54-infected leaves. This different dynamics of TP in grapevines infected with different phytoplasma genotypes can potentially be linked to the higher pathogenicity of M54. More pathogenic M54 caused a higher downregulation of phenolics biosynthesis at the beginning of the vegetative season and, on the one hand, a more severe upregulation at the end of the vegetative season. On the other hand, relative TP levels in M38-infected leaves did not vary between the second and third time points (Supporting Information 3).

**Table 1:** Content Total Phenolics and Groups of Phenolic Compounds in M38-Infected, M54-Infected, and Uninfected Grapevine Leaves at Three Time Points during the Development of Infection^a^

		**TP** (mg GAE/g DW)	**TF** (mg QE/g DW)	**TFl** (mg QE/g DW)	**TC** (mg CATE/g DW)	**TPAN** (mg CATE/g DW)	**TA** (mg C3GE/g DW)	**TPA** (mg CAE/g DW)	**THCA** (mg CAE/g DW)	**TA** (mg CATE/g DW)
June	M38-infected	66.40 ± 2.92 b	53.76 ± 1.42 b	2.96 ± 0.11 b	69.24 ± 1.29 a	30.32 ± 0.74 a	25.55 ± 0.89 b	32.46 ± 3.33 a	1.45 ± 0.12 b	45.31 ± 2.66 a
	M54-infected	52.81 ± 3.19 c	41.43 ± 0.49 c	2.39 ± 0.13 c	47.52 ± 1.04 c	20.56 ± 0.31 c	18.60 ± 1.10 c	27.60 ± 0.77 b	1.13 ± 0.11 c	32.13 ± 2.84 b
	uninfected	70.21 ± 3.30 a	57.07 ± 1.41 a	4.34 ± 0.13 a	55.61 ± 1.77 b	28.31 ± 1.35 b	31.97 ± 0.89 a	31.80 ± 2.81 a	2.52 ± 0.10 a	47.26 ± 2.93 a
August	M38-infected	53.26 ± 4.20 a	43.63 ± 2.45 a	2.36 ± 0.14 a	55.38 ± 2.07 b	22.26 ± 0.66 a	16.19 ± 1.17 b	29.00 ± 3.40 a	0.93 ± 0.15 a	33.32 ± 4.51 a
	M54-infected	49.83 ± 3.04 b	42.07 ± 1.05 a	2.05 ± 0.06 b	57.58 ± 1.75 a	22.36 ± 0.85 a	17.97 ± 2.09 a	27.07 ± 3.12 a	0.70 ± 0.88 b	33.51 ± 3.19 a
	uninfected	41.49 ± 2.29 c	38.46 ± 1.15 b	2.12 ± 0.22 b	31.25 ± 1.14 c	16.80 ± 0.96 b	17.29 ± 0.61 ab	29.38 ± 1.24 a	0.78 ± 0.20 b	22.71 ± 1.22 b
September	M38-infected	57.19 ± 5.21 b	51.16 ± 1.32 b	3.07 ± 0.15 a	59.26 ± 2.06 b	23.34 ± 0.75 b	16.80 ± 0.64 b	33.96 ± 1.42 a	1.29 ± 0.12 a	36.45 ± 3.15 b
	M54-infected	66.79 ± 2.26 a	58.40 ± 1.49 a	3.04 ± 0.10 a	70.05 ± 2.31 a	33.31 ± 1.56 a	27.73 ± 3.15 a	34.39 ± 3.69 a	1.23 ± 0.10 a	44.32 ± 1.50 a
	uninfected	44.23 ± 1.05 c	38.03 ± 1.25 c	2.62 ± 0.14 b	27.88 ± 0.92 c	14.38 ± 0.49 c	13.31 ± 1.14 c	32.08 ± 2.13 a	1.17 ± 0.17 a	25.67 ± 1.51 c

a Values represent mean ± standard deviation of three replicates with three technical replicates each. Different letters indicate a significant difference between the samples at a corresponding time point (ANOVA, Duncan test, *p* ≤ 0.05). TP = total phenolics; TF = total flavonoids; TFl = total flavonols; TC = total catechins; TPAN = total proanthocyanidins; TA = total anthocyanins; TPA = total phenolic acids; THCA = total hydroxycinnamic acids; TT = total tannins; DW = dry weight.

### 3.1 Impact on Phenolic Acids

**Figure 1:**
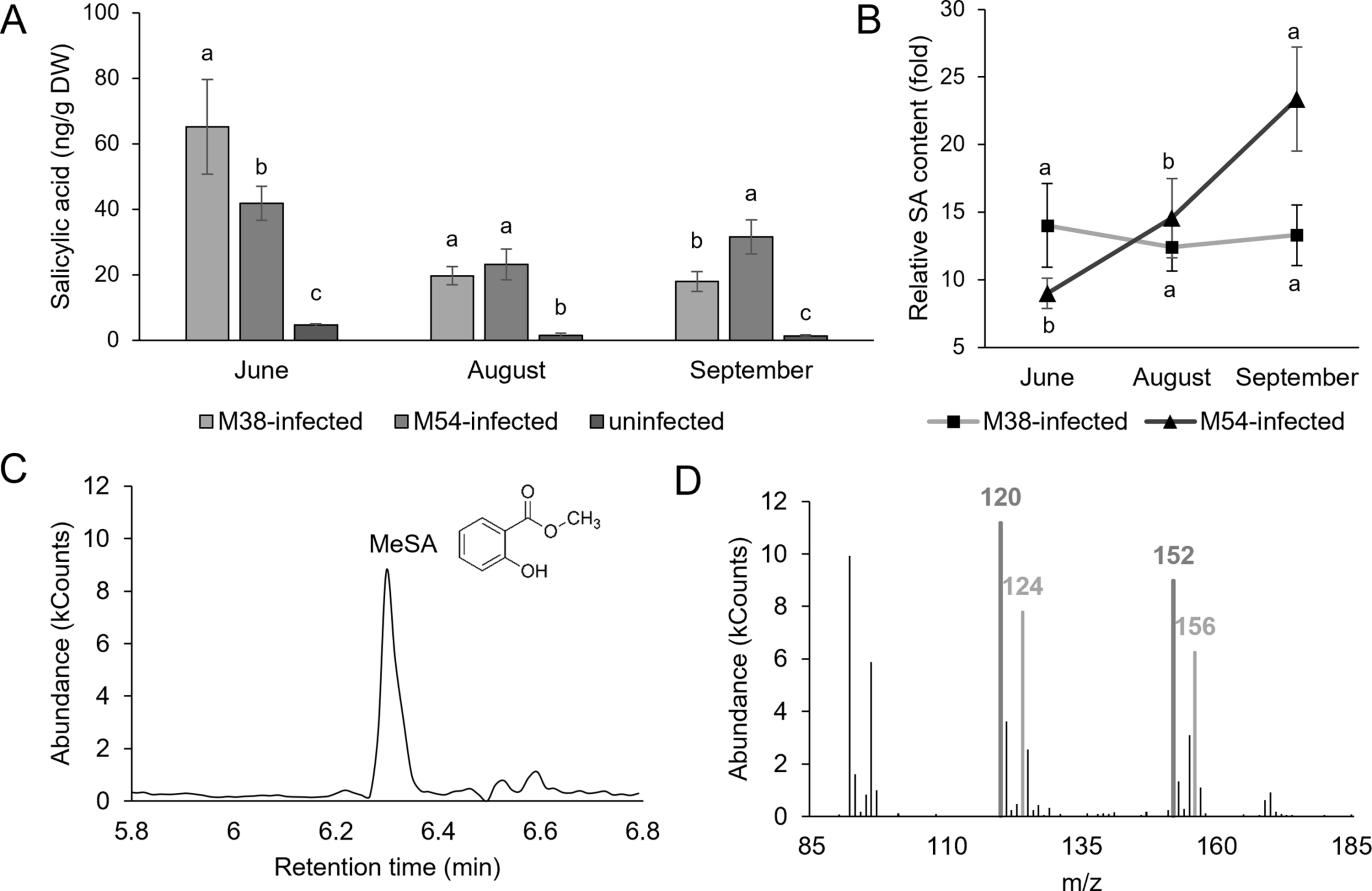
(A) Content of salicylic acid (SA) in M38-infected, M54-infected, and uninfected grapevine leaves at three time points during the development of infection. Values represent mean ± standard deviation of three replicates. Different letters indicate a significant difference between the samples at a corresponding time point (ANOVA, Duncan test, *p* ≤ 0.05). DW = dry weight. (B) Relative content of SA in M38- and M54-infected grapevine leaves at three time points, in relation to the corresponding controls. Values are ratios and represent mean ± standard deviation of three replicates. Different letters indicate a significant difference between different time points for the corresponding samples (ANOVA, Duncan test, *p* ≤ 0.05). (C) Representative obtained gas chromatogram recorded at *m*/*z* 120. Labeled peak at 6.299 min corresponds to the derivatized SA (methyl salicylate, MeSA); its molecular structure is indicated. (D) Mass spectrum recorded at 6.299 min. Marked are quantification ion 120 and molecular ion 152 of MeSA (dark gray), and quantification ion 124 and molecular ion 156 of heavy-labeled internal standard MeSA-D_4_ (light gray).

Phenolic acids are one of the main classes of phenolic compounds in grapevine.^31^ As their biosynthesis is at the beginning of the phenylpropanoid pathway, an impact on their content in infected leaves was expected. However, total phenolic acids (TPA) were lower in M54-infected leaves (Table 1) only at the first time point, presumably because of the downregulation observed for TP as well, whereas there was no difference further on during disease progression. Although TPA seemed not to be involved in response to the FDp infection, chromatographic analyses revealed that individual phenolic acids were affected by the FDp infection. Particularly, levels of SA were strongly elevated in infected leaves (Figure 1), with no exception. SA is a key signaling molecule in the plant defense response against pathogens.^18^ The involvement of SA in plant immunity responses in various pathosystems has been recognized,^17^ and its role is also demonstrated in this case, due to SA content being the most influenced parameter by FDp infection. The highest absolute levels of SA were at the first time point, reflecting the role of SA as an early response to infection as part of both PAMP-triggered immunity (PTI) and effector-triggered immunity (ETI). It has been reported that BNp could potentially trigger SA-dependent systemically acquired resistance (SAR) in grapevine leaves.^32^ Previous studies^11^ also described the increased levels of SA in FDp-infected grapevine, as well as the increase in expression of PATHOGENESIS-RELATED (*PR-2* and *PR-5*) genes, which are induced by the SA-mediated signaling. At the first time point, SA was the highest in M38-infected leaves; at the second time point, SA content was similar between M38- and M54-infected leaves; and at the third time point, M54-infected leaves had the highest SA levels. Similarly, TP was higher in M38-infected leaves at the first time point, but at the third, TP was higher in M54-infected leaves. Overall, differences between infected and uninfected leaves were more pronounced for SA, signifying a more specific response in contrast to the increase of TP. Similar trends between TP and SA could potentially point to SA biosynthesis as a response to FDp infection in FDp-infected leaves primarily from the phenylalanine ammonia lyase (PAL)-mediated phenylpropanoid pathway (and not the isochorismate pathway), which is a pathway leading to the synthesis of other phenolic compounds. However, this is only indirect evidence and must be experimentally tested. Even though SA is pronouncedly induced as a response to FDp, results for BNp-infected grapevine revealed that SA-mediated responses are ineffective in conferring resistance to BNp and actually inhibited the activation of jasmonate-mediated defenses, which appeared to be activated in recovered grapevine.^33^ This could also be the case for FDp-infected grapevine. Jasmonic acid (JA) is reported to potentially have a protective role against FDp infection.^34^ Furthermore, it is known that these two pathways often act antagonistically in different plant-pathogen systems.^35−37^ Therefore, it could be that SA synthesis is induced by yet uncharacterized FDp effectors as a way to antagonize the activation of JA synthesis, as previously demonstrated for BNp.^33^

Hydroxycinnamic acids are a group of phenolic acids that are derivatives of cinnamic acid (CA). Similarly to TPA, total hydroxycinnamic acids (THCA) were the lowest in M54-infected leaves at the first time point and the highest in uninfected leaves (Table 1). However, in BNp-infected leaves, THCA was reported to be increased compared to the uninfected plants at the beginning of the vegetative season,^29^ potentially because FDp has a more aggressive impact on THCA biosynthesis than BNp. At the first and second time points, M54-infected leaves had lower THCA, pointing to its higher impact on their biosynthesis. At the third time point, there was no difference in THCA between infected and uninfected leaves, with the same results reported for BNp-infected leaves at the end of the vegetative season.^29^ This points to a normalization in HCA biosynthesis in leaves infected with both genotypes.

CA is the product of the first step of the phenylpropanoid pathway, catalyzed by PAL. An increase in the expression of the *PAL* gene and PAL activity was previously observed in BNp-infected grapevine.^29,38^ However, a higher content of CA in infected leaves was not observed in our study but a statistically significant reduction in CA content at the first time point was measured (Table 2). This could be due to CA being an intermediate in the phenylpropanoid pathway and therefore redirected to the biosynthesis of other phenolic compounds (for example, SA, which was increased more than 10-fold). Interestingly, the highest PAL activity in BNp-infected grapevine was recorded in September by a previous study,^29^ the same period when the highest relative CA content was recorded in the present study, pointing to the increase of PAL activity potentially being the reason of the highest relative CA content in September in FDp-infected grapevine leaves. The CA content highly correlated with THCA (*r* = 0.97), pointing to hydroxycinnamic acid metabolism being dependent on CA levels in FDp-infected leaves. Also, CA highly correlated with TP (*r* = 0.76) but also with total flavonoids (*r* = 0.68), total flavonols (*r* = 0.93), total anthocyanins (*r* = 0.82), and total tannins (*r* = 0.72) (Supporting Information 4), revealing that impact on CA content potentially influences the entire phenylpropanoid pathway, because of the central role of CA in the biosynthesis of all phenolic compounds (Supporting Information 5). *p*-Coumaric acid (*p*-CA) is synthesized from CA by 4-cinnamic acid hydroxylase (4CH). Contrary to CA, *p*-CA content was increased in FDp-infected leaves at all time points, being higher in M38-infected leaves at the second time point and M54-infected leaves at the third time point. This could be due to its potential defensive functions in FDp-infected grapevine other than being an intermediate for the biosynthesis of other phenolics. Ferulic acid (FA), however, followed the same dynamics in M38- and M54-infected leaves as CA, supported also by the correlation (*r* = 0.82) between their content. Only at the third time point for both infected groups and at the second time point for M38-infected leaves was its content increased in infected leaves. This results point to its potential role in FDp-infected grapevine at the end of the vegetative season or could be just a consequence of the general upregulation of the phenylpropanoid pathway at the end of the vegetative season, as similar trends were observed for TP.

**Table 2:** Content of Individual Phenolic Compounds in M38-Infected, M54-Infected, and Uninfected Grapevine Leaves at Three Time Points during the Development of Infection^a^

		**cinnamic acid** (μg/g DW)	*p***-coumaric acid** (μg/g DW)	**ferulic acid** (μg/g DW)	**quercetin** (mg/g DW)	**myricetin** (μg/g DW)	**kaempferol** (μg/g DW)	**epicatechin** (mg/g DW)	**resveratrol** (μg/g DW)
June	M38-infected	765.70 ± 6.92 b	183.15 ± 6.48 a	172.14 ± 9.07 a	12.43 ± 0.04 b	1282.15 ± 19.61 a	1041.02 ± 1.55 a	6.62 ± 0.07 b	523.97 ± 20.48 c
	M54-infected	640.27 ± 6.52 c	193.79 ± 18.54 a	134.29 ± 6.33 b	9.92 ± 0.24 c	777.37 ± 22.31 b	667.42 ± 5.62 b	12.74 ± 0.83 a	582.31 ± 12.34 b
	uninfected	1013.28 ± 4.89 a	106.72 ± 1.69 b	175.62 ± 9.42 a	15.26 ± 0.30 a	1231.46 ± 22.97 a	579.10 ± 8.22 c	3.33 ± 0.28 c	922.51 ± 5.65 a
August	M38-infected	502.70 ± 5.56 a	105.36 ± 7.45 a	139.39 ± 1.73 a	12.88 ± 0.34 a	963.45 ± 12.37 a	867.15 ± 3.25 a	16.54 ± 0.71 a	288.51 ± 7.43 b
	M54-infected	499.77 ± 21.15 a	79.11 ± 1.10 b	122.93 ± 3.27 b	11.49 ± 0.14 b	902.16 ± 12.76 b	434.21 ± 5.92 c	17.16 ± 0.98 a	279.40 ± 7.39 b
	uninfected	521.56 ± 24.48 a	66.16 ± 1.83 c	119.26 ± 1.12 b	11.50 ± 0.50 b	683.61 ± 7.56 c	640.99 ± 9.43 b	18.00 ± 2.56 a	330.40 ± 13.00 a
September	M38-infected	601.38 ± 15.99 b	103.53 ± 3.11 b	142.20 ± 3.23 b	17.54 ± 0.29 a	1068.36 ± 28.81 b	1979.87 ± 40.21 a	24.94 ± 0.36 b	331.35 ± 2.90 b
	M54-infected	687.12 ± 19.97 a	124.34 ± 3.75 a	160.89 ± 3.35 a	17.06 ± 0.33 a	1430.06 ± 40.43 a	1431.782 ± 5.44 b	18.62 ± 0.83 c	370.76 ± 8.16 a
	uninfected	646.17 ± 21.29 ab	83.51 ± 1.57 c	124.92 ± 1.38 c	12.30 ± 0.21 b	702.87 ± 37.45 c	770.11 ± 5.00 c	33.79 ± 0.28 a	351.61 ± 14.90 ab

a Values represent mean ± standard deviation of three replicates. Different letters indicate a significant difference between the samples at a corresponding time point (ANOVA, Duncan test, *p* ≤ 0.05). DW = dry weight.

### 3.2 Involvement of Flavonoids in FDp Infection

The role of flavonoids during the phytoplasma infection has been studied the most out of all specialized metabolites.^2^ Previous studies have reported that grapevines infected with BNp showed both an upregulation of gene expression related to flavonoid production and increased activity of enzymes involved in flavonoid synthesis.^29,39^ The same was also observed for flavanone-3-hydroxylase in FDp-infected grapevine.^11^ At the first time point, total flavonoids (TF) were significantly decreased in infected leaves compared to the uninfected leaves, with M54-infected having the lowest TF. However, at the second and third time points, TF values were higher in leaves infected with both genotypes. Interestingly, the TF content correlated with the content of all major groups of flavonoids (flavonols, catechins, proanthocyanidins, and anthocyanins) and also individual flavonoids (quercetin and myricetin), indicating similar trends in all branches of the flavonoid pathway and pointing to a general response of this pathway to the FDp infection. The trends for total flavonols (TFl) were slightly different than for TF, because the TFl content in M54-infected leaves at the second time point was similar to the uninfected leaves and no difference between TFl between M38- and M54-infected leaves at the third time point was observed. However, the relative dynamics for TFl and TF were the same in leaves infected with both genotypes M38 and M54 (Supporting Information 3). Also, in BNp-infected grapevine leaves, higher TFl were observed early in the vegetative season only,^29^ indicating a higher impact of FDp infection to flavonol metabolism in grapevine.

Quercetin, myricetin, and kaempferol were previously identified as the main flavonol aglycones in grapevine leaves,^40^ and this was confirmed in this study. The quercetin content exhibited the same trend as TFl, probably due to being the most abundant flavonol identified. Another study tested the potential of exogenous quercetin to induce the recovery of ‘*Ca.* P. asteris’-infected *Catharanthus roseus* (L.) G. Don shoots grown *in vitro.* However, no impact on phytoplasma infection was observed.^41^ Therefore, its accumulation probably does not have direct defensive properties against FDp. The myricetin content changed in a manner similar to quercetin. However, FDp infection changed the content of kaempferol in a different manner compared to quercetin and myricetin. At the first time point, the lowest kaempferol content was in uninfected leaves, pointing to an increase in the kaempferol content being an early response to FDp infection. A study on virus-infected *Arabidopsis thaliana* also discussed the role of kaempferol in early responses against infection, even preceding the response of SA.^42^ Considering that both viruses and phytoplasmas are retained in the symplast, and both are essentially intracellular parasites, plant responses to the infection could be similar. Interestingly, in both M38- and M54-infected leaves, the relative kaempferol content was the lowest at the second time point, in contrast to the quercetin and myricetin levels, which had the lowest relative content at the first time point (Supporting Information 6).

The content of total catechins (TC), another class of flavonoid compounds, followed the patterns observed for TF. However, the only individual catechin identified, epicatechin, had a higher content in infected leaves at the first time point, similar levels between infected and uninfected leaves at the second, and lowest levels in infected leaves at the third time point. Epicatechin is synthetized from cyanidin by anthocyanidin reductase (ANR).^43^ However, cyanidin is a shared intermediate, which can also be converted to an anthocyanin, cyanidin-3-*O*-glucoside, by the activity of flavonoid 3-*O*-glucosyltransferase (F3GT).^44^ It could be possible that those enzymes are in competition and higher expression of F3GT caused the decrease in epicatechin content by depleting the substrate for ANR, explaining the negative correlation between epicatechin and total anthocyanins (*r* = −0.75). The content of total proanthocyanidins (condensed tannins; catechin oligomers) followed the trend of TC, and a correlation between their contents was observed (*r* = 0.92). Considering that proanthocyanidins are direct polymers of catechins, such a link between their content was expected. Interestingly, TC levels between M38- and M54-infected leaves varied at the second time point, where M38-infected leaves had lower TC, but levels of proanthocyanidins were the same between M38- and M54-infected leaves. Catechin monomers seem to be more susceptible to content change than catechin oligomers, which is to be expected since proanthocyanidins are the more stable (storage) forms of catechins. Also, the levels of total tannins (TT), which include proanthocyanidins and hydrolyzable tannins (nonflavonoid compounds), strongly correlated with proanthocyanidin content (*r* = 0.94). Together with the dynamics observed for TT, these results indicate that the proanthocyanidin portion of tannins are the main tannins susceptible to FDp-induced changes and hydrolyzable tannins being either affected with similar dynamics or unaffected by FDp infection in grapevine.

Although it was not present in FDp-infected ‘Pinot gris’, leaf reddening is a common symptom of FDp infection in some grapevine varieties and it is attributed to anthocyanin accumulation.^45^ Therefore, some increase in the total anthocyanin (TA) content in infected leaves was expected. General dynamics of TA content was the same as for TF; however, a higher content of TA in infected leaves compared to the uninfected ones was only observed at the third time point. It was previously reported for healthy grapevine leaves that the expression of genes related to anthocyanin biosynthesis reduces to the end of the vegetative season,^46^ with our results for uninfected leaves confirming these results. However, in infected leaves, the highest anthocyanin content, particularly in M54-infected leaves, was at the third time point, late in the vegetative season, confirming that FDp infection completely modifies the normal seasonal dynamics of anthocyanins. Considering that many plants accumulate anthocyanins as a response to the chlorophyll loss,^47^ anthocyanin accumulation could protect leaves from potentially damaging light levels due to the FDp-induced chlorophyll reduction. Interestingly, in BNp-infected grapevine, the only anthocyanin increased in infected leaves was cyanidin 3-*O*-glucoside.^15^ This further adds to the previous hypothesis about the increase in expression of F3GT, which led to the increase of cyanidin 3-*O*-glucoside and decrease in epicatechin. Also, its content was significantly higher in September compared to July in BNp-infected leaves,^15^ with similar dynamics for total anthocyanins observed in FDp-infected leaves in this study. Therefore, the hypothesis is that cyanidin 3-*O*-glucoside is also the main anthocyanin, whose increase in content is induced by FDp infection.

### 3.3 Resveratrol Content Is Reduced in FDp-Infected Grapevine

Stilbenoids are a class of phenolic compounds derived from the main phenylpropanoid pathway and are important in plants as phytoalexins, and their production is known to be induced by both biotic and abiotic stresses. In particular, resveratrol is a main stilbenoid synthesized in grapevine.^48^ Resveratrol content was never higher in FDp-infected leaves compared to the uninfected ones. However, a previous study^15^ recorded a higher content of resveratrol-glucoside in September in BNp-infected grapevine leaves compared to the uninfected leaves. Similar to results of the present study, a lower content of resveratrol is observed in FDp-infected leaves compared to the uninfected ones, along with a lower expression of stilbene synthase, involved in resveratrol synthesis.^10^ However, in recovered plants, both resveratrol content and stilbene synthase expression were increased,^10^ pointing to the role of resveratrol in recovery against FDp and also FDp-mediated repression of stilbenoid synthesis. Due to the resveratrol content being lower in M38-infected leaves than in M54-infected leaves at the first and third time points, infection with FDp genotype M38 could potentially have a higher effect on resveratrol content, therefore hindering the potential protective role of resveratrol against FDp.

### 3.4 Phenolics Accumulation Affects the Antioxidative Status of FDp-Infected Grapevine

**Figure 2:**
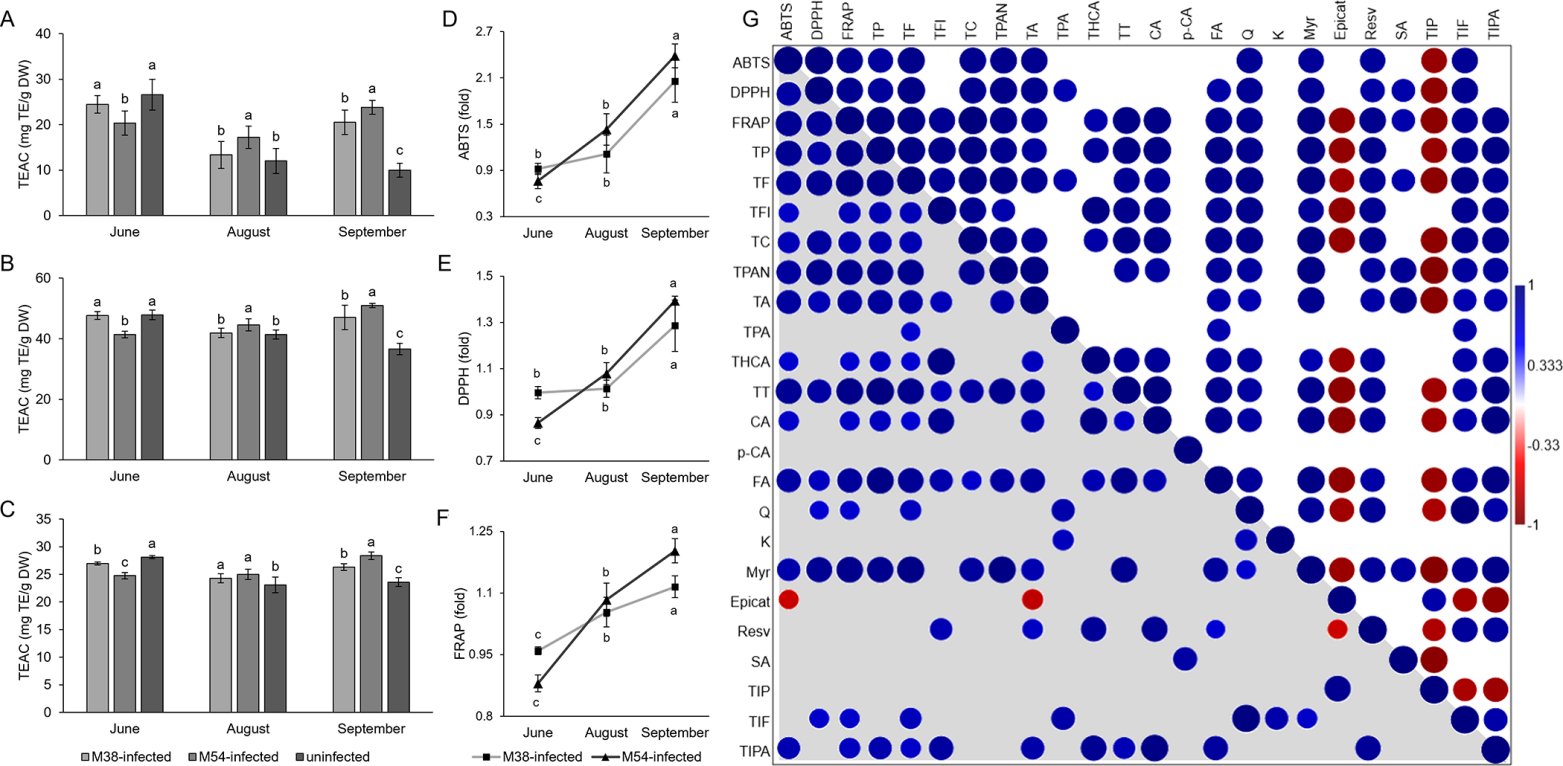
Results of antioxidative capacity assays and correlations between antioxidative capacity and phenolic levels. Antioxidative capacity of grapevine leaf extracts measured by (A) ABTS, (B) DPPH, and (C) FRAP assays of M38-infected, M54-infected, and uninfected grapevine leaves at three time points during the development of infection. Values represent mean ± standard deviation of three replicates with three technical replicates each. Different letters indicate a significant difference between the samples at a corresponding time point (ANOVA, Duncan test, *p* ≤ 0.05). TEAC = Trolox equivalent antioxidative capacity, TE = Trolox equivalents, DW = dry weight. (D–F) Antioxidative capacity of M38- and M54-infected grapevine leaf extracts at three time points, in relation to the corresponding controls measured by (D) ABTS, (E) DPPH, and (F) FRAP assays. Values are ratios and represent mean ± standard deviation of three replicates with three technical replicates each. Different letters indicate a significant difference between different time points for the corresponding samples (ANOVA, Duncan test, *p* ≤ 0.05). (G) Pearson’s correlations between absolute (lower triangle in gray) and relative (upper triangle in white) values of metabolic parameters and antioxidative capacity. Statistically significant correlations (*p* ≤ 0.05) are marked. Numerical values obtained from correlation analyses are provided in Supporting Information 5. Abbreviations: ABTS, DPPH, FRAP = antioxidative capacity assays; TP = total phenolics; TF = total flavonoids; TFl = total flavonols; TC = total catechins; TPAN = total proanthocyanidins; TA = total anthocyanins; TPA = total phenolic acids; THCA = total hydroxycinnamic acids; TT = total tannins; CA = cinnamic acid; p-CA = *p*-coumaric acid; FA = ferulic acid; Q = quercetin; K = kaempferol; Myr = myricetin; Epicat = epicatechin; Resv = resveratrol; SA = salicylic acid; TIP = total identified phenolics; TIF = total identified flavonoids; TIPA = total identified phenolic acids.

Considering that phenolic compounds are known to have a role as antioxidants in plants, it was argued that induction of their synthesis in FDp-infected grapevine could limit oxidative damage,^9^ which is primarily caused by the reduction of photosynthetic capacity of infected grapevine leaves. Therefore, ABTS, DPPH, and FRAP assays were used to assess the antioxidative capacity of grapevine leaf extracts. Levels of antioxidative capacity measured by all three assays were mutually correlated, confirming the validity of the results. Also, antioxidative capacity was also highly correlated with TP but also with most groups and individual phenolic compounds, confirming that phenolics are the main mediators of antioxidative capacity in FDp-infected leaves (Figure 2). Lower antioxidative capacity in M54-infected leaves at the first time point (measured by ABTS and DPPH), and for both M38- and M54-infected leaves as measured by FRAP, as well as in M38-infected leaves at the second time point (as measured by ABTS and DPPH) compared to the uninfected leaves points to a downregulation in biosynthesis of key antioxidants at the beginning of the vegetative season in FDp-infected plants. For the third time point, all three assays revealed the highest antioxidative capacity in M54-infected leaves, followed by M38-infected leaves, pointing to the induction of biosynthesis of key antioxidants to the end of the vegetative season.

**Figure 3:**
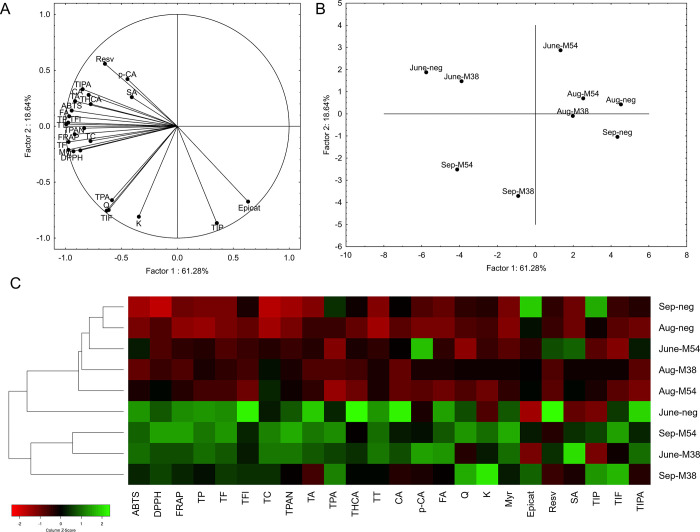
Results of chemometric analyses. Principal component analysis (PCA) based on the assessed metabolic parameters: (A) loading plot of the measured parameters and (B) score plot separating the groups based on the measured parameters. (C) Heatmap displaying the relationship between tested parameters, with hierarchical clustering based on Euclidian distance applied to visualize the relationship between groups. June, Aug, Sep = June, August, and September time points, respectively. M38 = M38-infected leaves, M54 = M54-infected leaves, neg = uninfected leaves. ABTS, DPPH, FRAP = antioxidative capacity assays; TP = total phenolics; TF = total flavonoids; TFl = total flavonols; TC = total catechins; TPAN = total proanthocyanidins; TA = total anthocyanins; TPA = total phenolic acids; THCA = total hydroxycinnamic acids; TT = total tannins; CA = cinnamic acid; p-CA = *p*-coumaric acid; FA = ferulic acid; Q = quercetin; K = kaempferol; Myr = myricetin; Epicat = epicatechin; Resv = resveratrol; SA = salicylic acid; TIP = total identified phenolics; TIF = total identified flavonoids; TIPA = total identified phenolic acids.

Principal component analysis (Figure 3) visualized the relationship between examined samples, based on the levels of groups and individual phenolic compounds and antioxidative capacity. M38-infected and uninfected leaves from the first time point grouped together; however, M54-infected leaves were separated from the other two, pointing to a higher impact of M54 to the phenolic profile in grapevine at the first time point. The same conclusion can be drawn from the number of parameters that were decreased in comparison to the control, namely, 14 for M54-infected leaves and nine for M38-infected leaves (Supporting Information 7). At the August time point, all three examined samples clustered together, potentially due to the normalization of the phenolic content from the first time point, where a downregulation in infected leaves was observed. This is also supported by the lower number of parameters reduced in comparison to that at the June time point. At the September time point, both infected groups separated from the uninfected leaves, revealing the high modification of the phenolic profile in infected leaves. Particularly, the M54-infected leaves separated more distantly from the uninfected leaves. This is interesting considering that M54-infected leaves had the highest number of parameters with the highest levels of the three groups at the September time point. Although both infected groups had 14 parameters increased each, M54-infected leaves had the most parameters with the highest phenolic content, pointing to the more severe upregulation of phenolic metabolism, as well as consequential increase in antioxidative capacity, in M54-infected leaves at the end of the vegetative season.

To conclude, in comparison with previous metabolomic and transcriptomic analyses of FDp-infected grapevine, our results reveal the upregulation of the phenylpropanoid pathway as a result of FDp infection. Accumulation trends for the groups of the main branches of the phenylpropanoid pathway, as well as most individual identified phenolic compounds, revealed that their content increases throughout the development of infection. However, as previously argued,^9^ increased phenolic content probably does not have a protective role against FDp infection, considering that phenolic compounds are mostly compartmentalized in the vacuole and cell wall, spatially separated from phloem-restricted phytoplasmas. High accumulation of phenolic compounds in infected leaves highly increased the antioxidative capacity of grapevine leaves, as measured by ABTS, DPPH, and FRAP assays. This increase in antioxidative compounds content could quench the grapevine-induced ROS produced as defense molecules against FDp, considering that the role of ROS in grapevine defense and recovery against FDp was previously recognized.^49,50^ Also, accumulation of flavonoids and related compounds could protect infected leaves, whose photosynthetic pigment content is highly reduced, from UV-induced stress, therefore postponing the senescence of leaves and prolonging the period for phytoplasmas to be transmitted to the vector. Considering that most of the content of most of the classes of phenolic compounds are increased in infected leaves, induction of this pathway could probably be traced to the potential FDp effectors impacting the expression of stem genes of the phenylpropanoid pathway. Such effectors targeting *PAL* and other stem phenolic biosynthesis genes were previously described in different plant pathogens. So far, effectors SnTox3 from *Parastagonospora nodorum* and ToxA and ToxB from *Pyrenophora tritici-repentis* have been demonstrated to induce *PAL* expression.^51^ Our results further demonstrate that this could also be the case for FDp as a potential way to promote phytoplasma transmission. This could aid future studies to identify the presence of similar effectors in FDp genomes, as well as their targets in plant hosts. However, the upregulation of phenolic biosynthesis was observed only later in the development of the infection. At the first time point, the content of most compounds was lower in infected leaves in comparison to the uninfected ones. This was particularly visible in M54-infected leaves, which impacted phenolic metabolism more severely but also activated it more severely at the third time point, considering the highest levels of phenolic compounds recorded in M54-infected leaves at the September time point. Potentially, at the beginning of the vegetative season, when the phytoplasma titer is low in host plants, phenolics could act defensively against FDp. Therefore, their biosynthesis could be downregulated at the beginning of the vegetative season for this reason. Reasons for the differences in severity of the two genotypes investigated should be further explored by whole-genome analysis of the strains studied in this work. Further studies should focus on exploring the mechanisms of FDp-related induction of phenolic metabolism, particularly SA, and repression of synthesis of compounds that were previously related to recovery from FDp, particularly resveratrol, whose content in this study was reduced in infected leaves, as well as identification of putative FDp effectors involved in the modification of those pathways. Our results demonstrate variable pathogenicity between FDp strains, with the more aggressive genotype M54 modulating the host metabolism in a more severe way than did the less aggressive M38. This shows the need for future studies to take into account the highly plastic phytoplasma genomes and their consequences in spread and pathogenicity of different strains. This also points to the potential need for developing different management strategies of FDp to combat these highly versatile plant pathogens.

## Supporting Information Available

(Supplementary file 1) detailed description of spectrophotometric assays used for the determination of total phenolic content and content of the groups of phenolic compounds; (Supplementary file 2) representative chromatograms obtained after the HPLC analysis of grapevine leaves extracts, recorded at 254, 280, 310, and 360 nm with peaks labeled with corresponding identified compounds; (Supplementary file 3) relative content of total phenolics and groups of phenolic compounds of M38- and M54-infected grapevine leaves at three time points, in relation to the corresponding controls; (Supplementary file 4) Pearson’s correlation coefficients (*r*) between the absolute and relative values of tested parameters; (Supplementary file 5) schematic representation of the main phenylpropanoid pathway with indicated main branches of phenolic metabolism; (Supplementary file 6) relative content of individual phenolic compounds of M38- and M54-infected grapevine leaves at three time points, in relation to the corresponding controls; (Supplementary file 7) number of increased, decreased, and resistant (no difference) parameters for M38- and M54-infected leaves in comparison to the corresponding controls for three time points (June, August, September), and number of parameters that had the highest values at each time point (June, August, September) for every analyzed group (PDF)

## Abbreviations

**BNp**: *bois noir* phytoplasma (*Ca.* ‘P. solani’)
***Ca.***: *Candidatus*
**CA**: cinnamic acid
**DW**: dry weight
**FA**: ferulic acid
**FDp**: *flavescence dorée* phytoplasma
**PAL**: phenylalanine-ammonium lyase
**p-CA**: *p*-coumaric acid
**ROS**: reactive oxygen species
**SA**: salicylic acid
**TA**: total anthocyanins
**TC**: total catechins
**TF**: total flavonoids
**TFl**: total flavonols
**TP**: total phenolics
**TPA**: total phenolic acids
**TPAN**: total proanthocyanidins
**TT**: total tannins
