# Supplementary material for: Flavescence Dorée Strain-Specific Impact on Phenolic Metabolism Dynamics in Grapevine (Vitis vinifera) throughout the Development of Phytoplasma Infection
Source: J Agric Food Chem. 2023 Dec 19;72(1):189–99. doi: 10.1021/acs.jafc.3c06501 (PMC10786034; doi:10.1021/acs.jafc.3c06501)
Supplement: Supplementary file 1 — jf3c06501_si_003.pdf [file jf3c06501_si_003.pdf]

Supporting information for

***Flavescence dorée* strain-specific impact on phenolic metabolism dynamics in grapevine (*Vitis vinifera*) throughout the development of phytoplasma infection**

Dino DAVOSIR[a, b], Ivana ŠOLA[a], Jutta LUDWIG-MÜLLER[b], Martina ŠERUGA MUSIĆ[a, *]

[a] Department of Biology, Faculty of Science, University of Zagreb, Horvatovac 102a, 10000 Zagreb, Croatia; (ddavosir@stud.biol.pmf.hr (DD); ivana.sola@biol.pmf.hr (IŠ); martina.seruga.music@biol.pmf.hr (MŠM))

[b] Faculty of Biology, Technische Universität Dresden, Zellescher Weg 20b, 01217 Dresden, Germany (Jutta.Ludwig-Mueller@tu-dresden.de (JLM))

[*] Correspondence: martina.seruga.music@biol.pmf.hr; +385(0)1 4898 097

Supplementary file 1

Detailed description of spectrophotometric assays used for the determination of total phenolic content and content of the groups of phenolic compounds.

1.1. Determination of total phenolics content

For the determination of total phenolics content in grapevine leaves extracts, method according to Singleton et al. (1999) was used. Extracts (10 μL) were mixed with 790 μL of deionized water and 50 μL of Folin-Ciocaulteu (FC) reagent and the mixture was homogenized on a vortex mixer. Then, 150 μL of 1.88 M Na_2CO_3 were added and the mixture was homogenized and incubated for 30 min at 45 °C in an incubator. After the incubation, 200 μL of the sample were transferred in four technical replicates on a 96-well plate and the colour intensity was quantified by measuring the absorbance at 765 nm on a FLUOstar Optima microplate reader (BMG LABTECH, Ortenberg, Germany). As blank, 70% ethanol was used instead of the extract. The measured absorbance of the blank was subtracted from the absorbance of the samples and the total phenolics content in the samples was calculated indirectly as mg of gallic acid equivalents (GAE) per g of DW based on the calibration curve of standard gallic acid solutions of known concentrations (5-0.05 mg/mL).

1.2. Determination of flavonoid content

For the determination of total flavonoid content in grapevine leaves extracts, method according to Zhishen et al. (1999) was used. Extracts (70 μL) were mixed with 280 μL of deionized water and 21 μL of 5% $NaNO_2$ solution and the mixture was homogenized on a vortex mixer and incubated for 5 min at room temperature (RT). Then, 21 μL of 10% $AlCl_3$ was added, and the mixture was homogenized and incubated for 6 min at RT. After incubation, 140 μL of 1 M NaOH and 168 μL of deionized water were added. Afterwards, 200 μL of each sample were transferred in three technical replicates on a 96-well plate and the colour intensity was quantified by measuring the absorbance at 510 nm on a FLUOstar Optima microplate reader (BMG LABTECH, Ortenberg, Germany). As blank, 70% ethanol was used instead of the extract. The measured absorbance of the blank was subtracted from the absorbance of the samples and the total flavonoid content in the samples was calculated indirectly as mg of quercetin equivalents (QE) per g of DW, based on the calibration curve of standard quercetin solutions of known concentrations (1-0.0125 mg/mL).

1.3. Determination of catechin content

Catechin (flavan-3-ol) content in grapevine leaves extracts was evaluated using the method modified from Rusak et al. (2021). The extract was diluted with 70% ethanol (v/v) to 10 mg/mL and 280 μL of each diluted extract was mixed with 420 μL of DMACA (0.1% *p*-dimethylaminocinnamaldehyde in 1 M HCl, in methanol) solution. After mixing the samples on a vortex mixer, 200 μL of each sample were transferred in three technical replicates on a 96-well plate and the colour intensity was quantified by measuring the absorbance at 595 nm on a FLUOstar Optima microplate reader (BMG LABTECH, Ortenberg, Germany). As blank, 70% ethanol was used instead of the extract. The measured absorbance of the blank was subtracted from the absorbance of the samples and the total catechin content in the samples was calculated indirectly as mg of catechin equivalents (CE) per g of DW based on the calibration curve of standard catechin solutions of known concentrations (1-0.01 mg/mL).

1.4. Determination of proanthocyanidin content

Proanthocyanidin (condensed tannin) content was evaluated according to the method in Šamec et al. (2014). Away from light, 420 μL of vanillin (4%, w/v; dissolved in methanol) was mixed with 70 μL of the extract and 210 μL of HCl was added. The mixture was homogenized on a vortex mixer and incubated for 15 min at RT. After the incubation, 200 μL of each sample were transferred in three technical replicates on a 96-well plate and the colour intensity was quantified by measuring the absorbance at 495 nm on a FLUOstar Optima microplate reader (BMG LABTECH, Ortenberg, Germany). As blank, 70% ethanol was used instead of the extract. The measured absorbance of the blank was subtracted from the absorbance of the samples and the total catechin content in the samples was calculated indirectly as mg of catechin equivalents (CE) per g of DW, based on the calibration curve of standard catechin solutions of known concentrations (1-0.01 mg/mL).

1.5. Determination of anthocyanin content

Content of anthocyanins in grapevine leaves extracts was evaluated using the method modified from Tušek et al. (2016). A volume of 150 μL of each extract was mixed with 450 μL of 70% (v/v) ethanol and 50.4 μL of concentrated HCl. The samples were briefly mixed on a vortex mixer and incubated for 60 min in a Thermomixer (Eppendorf, Hamburg, Germany) at 80 °C and 300 rpm. After the incubation, 200 μL of each sample were transferred in three technical replicates on a 96-well plate and the colour intensity was quantified by measuring the

absorbance at 520 nm on a FLUOstar Optima microplate reader (BMG LABTECH, Ortenberg, Germany). As blank, 70% ethanol was used instead of the extract. The measured absorbance of the blank was subtracted from the absorbance of the samples and the total anthocyanin content in the samples was calculated indirectly as cyanidin-3-glucoside equivalents (C3GE) based on the molar extinction coefficient of 34300 M^{-1} cm^{-1} adapted for the measurement using the microplate reader and a molecular weight of 449.2 g mol^{-1}.

1.6. Determination of flavonol and hydroxycinnamic acid content

Flavonols and hydroxycinnamic acids content was estimated according to the method described in Howard et al. (2003). Extract (10 μL) was mixed with 40 μL of 70% (v/v) ethanol, 50 μL of HCl (1 g/L in 96% ethanol) and 910 μL of HCl (2 g/L in H_2O). After homogenizing the sample on a vortex mixer, 200 μL of each sample were transferred in four technical replicates on a 96-well plate and the colour intensity was quantified by measuring the absorbance at 320 nm for hydroxycinnamic acids and 360 nm for flavonols on a MultiSkan SkyHigh Microplate Spectrophotometer (Thermo Fisher Scientific, Waltham, USA). As blank, 70% ethanol was used instead of the extract. The measured absorbance of the blank was subtracted from the absorbance of the samples and the flavonol content in the samples was calculated indirectly as mg of quercetin equivalents (QE) per g of DW based on the calibration curve of standard quercetin solutions of known concentrations (1-0.00625 mg/mL). The hydroxycinnamic acid content in the samples was calculated indirectly as mg of caffeic acid equivalents (CAE) per g of DW based on the calibration curve of standard caffeic acid solutions of known concentrations (2-0.05 mg/mL).

1.7. Determination of phenolic acid content

Phenolic acids content was estimated using the method described in Šamec et al. (2014), with some modifications. Extract (70 μL) was mixed with 140 μL of 0.5 M HCl and 140 μL of Arnow reagent (10% $NaNO_2$, 10% Na_2MoO_2 in H_2O), 140 μL of 8.5% NaOH and 210 μL of H_2O. After homogenizing the sample on a vortex mixer, 200 μL of each sample were transferred in three technical replicates on a 96-well plate and the colour intensity was quantified by measuring the absorbance at 495 nm on a FLUOstar Optima microplate reader (BMG LABTECH, Ortenberg, Germany). As blank, 70% ethanol was used instead of the extract. The measured absorbance of the blank was subtracted from the absorbance of the samples and the phenolic acid content in the samples was calculated indirectly as mg of caffeic

acid equivalents (CAE) per g of DW based on the calibration curve of standard caffeic acid solutions of known concentrations (2-0.1 mg/mL).

1.8. Determination of tannin content

For the determination of tannins content, method according to Sangeetha & Vedasree (2012) was used. Away from light, 840 μL of deionized H_2O, 50 μL of FC reagent, 100 μL of 3.5% Na_2CO_3 and 10 μL of the extract was mixed and homogenized on a vortex mixer followed by an incubation for 30 min at RT. After the incubation, 200 μL of each sample were transferred in three technical replicates on a 96-well plate and the colour intensity was quantified by measuring the absorbance at 700 nm on a MultiSkan SkyHigh Microplate Spectrophotometer (Thermo Fisher Scientific, Waltham, USA). As blank, 70% ethanol was used instead of the extract. The measured absorbance of the blank was subtracted from the absorbance of the samples and the total catechin content in the samples was calculated indirectly as mg of catechin equivalents (CE) per g of DW based on the calibration curve of standard catechin solutions of known concentrations (2-0.05 mg/mL).

References

Howard, L. R., Clark, J. R., & Brownmiller, C. (2003). Antioxidant capacity and phenolic content in blueberries as affected by genotype and growing season. *Journal of the Science of Food and Agriculture, 83*(12), 1238–1247. https://doi.org/10.1002/jsfa.1532

Rusak, G., Šola, I., & Vujčić Bok, V. (2021). Matcha and Sencha green tea extracts with regard to their phenolics pattern and antioxidant and antidiabetic activity during in vitro digestion. *Journal of Food Science and Technology*, *58*(9), 3568–3578. https://doi.org/10.1007/s13197-021-05086-5

Sangeetha, R., & Vedasree, N. (2012). *In vitro* α-amylase inhibitory activity of the leaves of *Thespesia populnea*. *International Scholarly Research Network ISRN Pharmacology, 2012*. https://doi.org/10.5402/2012/515634

Singleton, V. L., Orthofer, R., & Lamuela-Raventós, R. M. (1999). Analysis of total phenols and other oxidation substrates and antioxidants by means of Folin-Ciocalteu reagent. *Methods in Enzymology, 299*, 152–178. https://doi.org/10.1016/S0076-6879(99)99017-1

Šamec, D., Bogović, M., Vincek, D., Martinčić, J., & Salopek-Sondi, B. (2014). Assessing the authenticity of the white cabbage (*Brassica oleracea* var. *capitata* f. alba) cv. “Varaždinski”

by molecular and phytochemical markers. *Food Research International*, *60*, 266–272. https://doi.org/10.1016/j.foodres.2013.07.015

Tušek, M., Curman, M., Babić, M., & Tkalec, M. (2016). Photochemical efficiency, content of photosynthetic pigments and phenolic compounds in different pitcher parts of Sarracenia hybrids. *Acta Botanica Croatica, 75*(2), 179–185. https://doi.org/10.1515/botcro-2016-0036

Zhishen, J., Mengcheng, T., & Jianming, W. (1999). The determination of flavonoid contents in mulberry and their scavenging effects on superoxide radicals. *Food Chemistry*, *64*(4), 555–559. https://doi.org/10.1016/S0308-8146(98)00102-2

Supplementary file 2

Representative chromatograms after the HPLC analysis of grapevine leaves extracts recorded at (A) 254 nm, (B) 280 nm, (C) 310 nm and (D) 360 nm with peaks labelled with corresponding identified compounds. mAU = milliabsorbance units, Myr = myricetin, Epicat = epicatechin, CA = cinnamic acid, p-CA = *p*-coumaric acid, FA = ferulic acid, Resv = resveratrol, Q = quercetin, K = kaempferol.

A

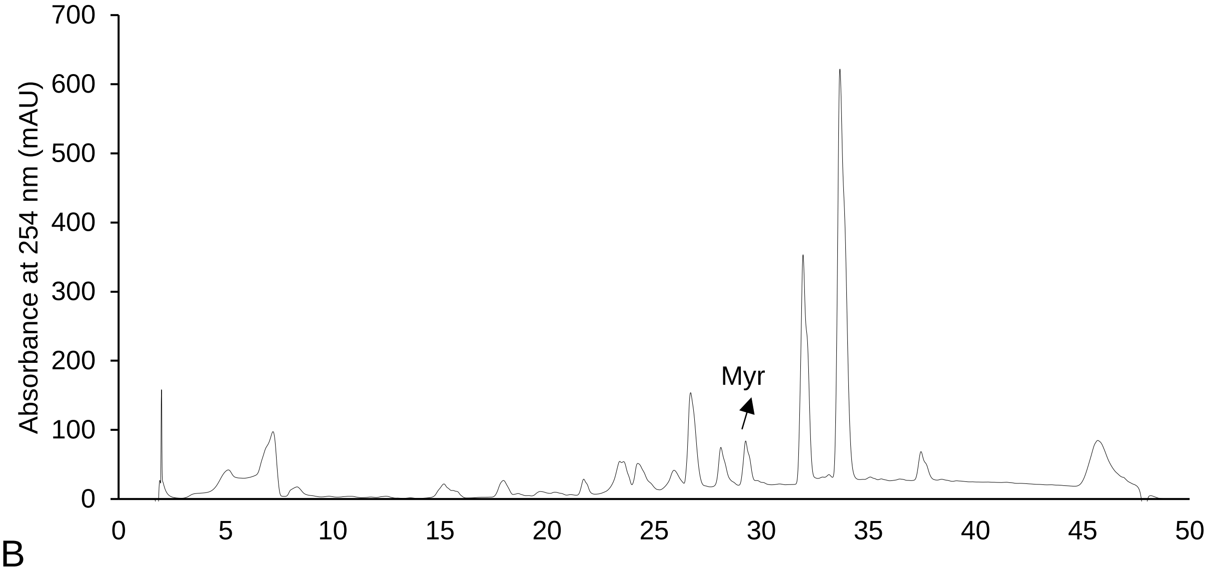

B

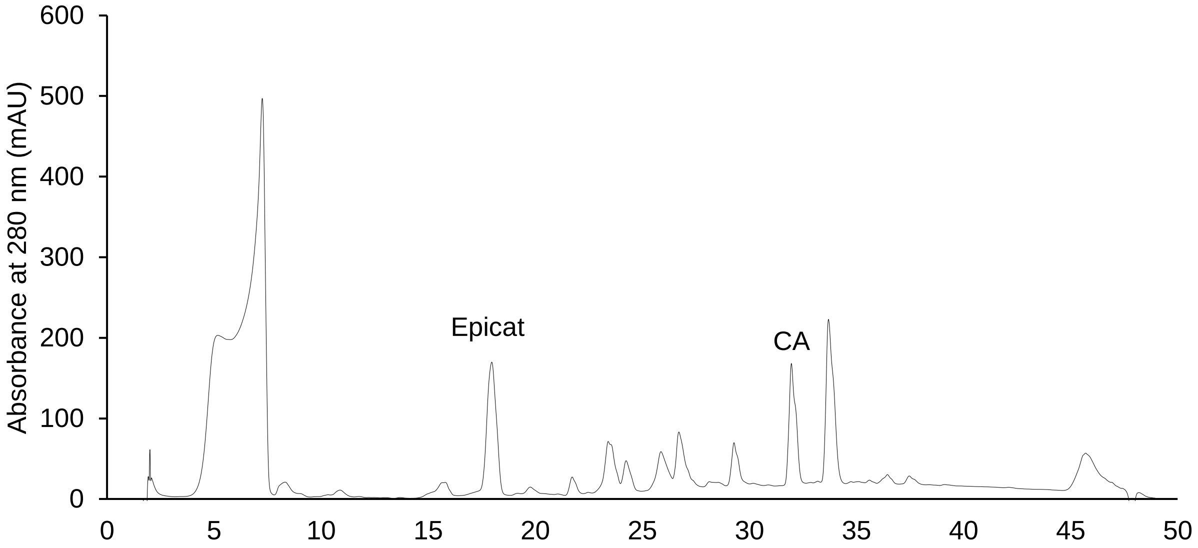

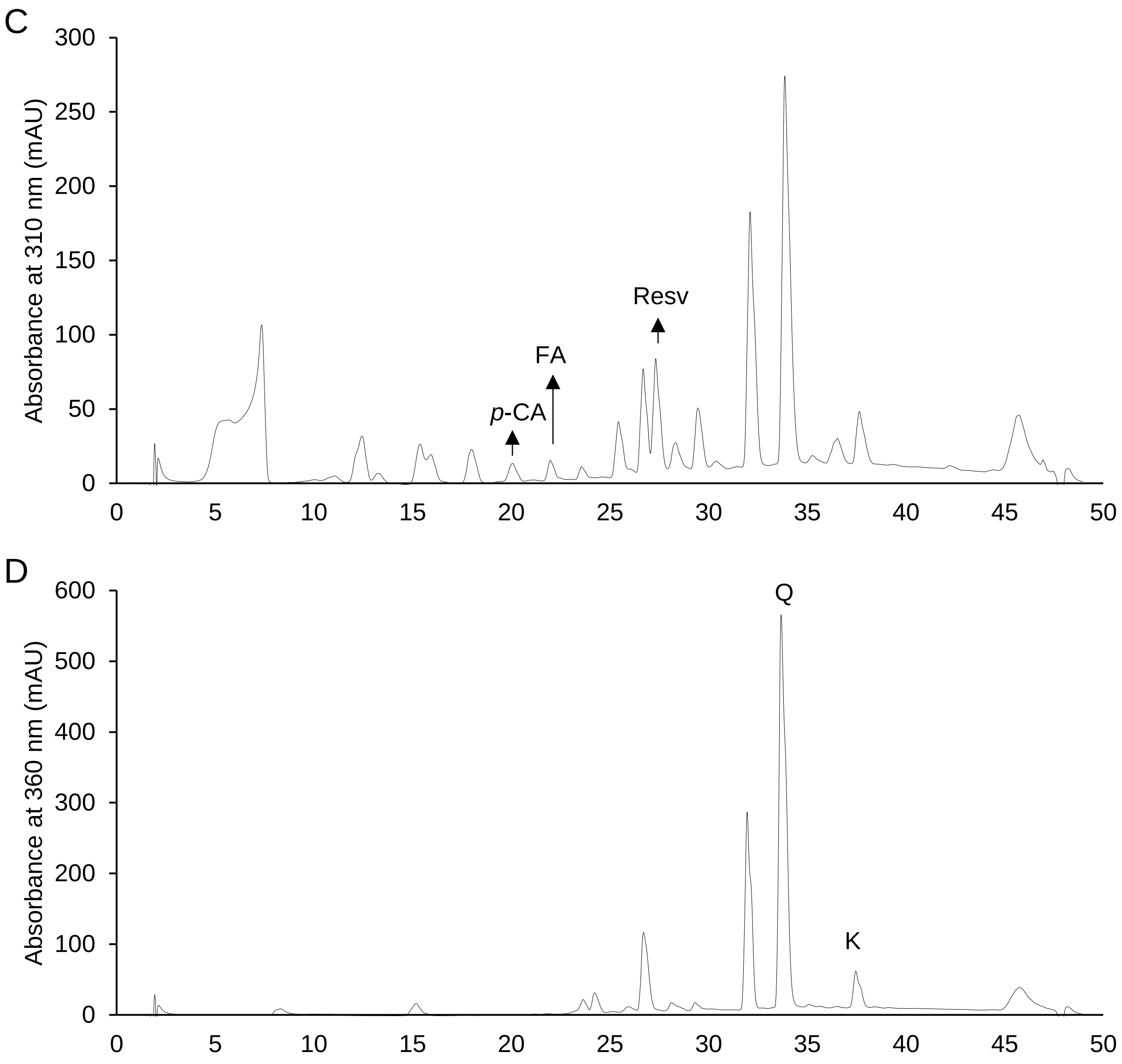
C
Absorbance at 310 nm (mAU)
300
250
200
150
100
50
0
p-CA
FA
Resv
0
5
10
15
20
25
30
35
40
45
50
D
Absorbance at 360 nm (mAU)
600
500
400
300
200
100
0
Q
K
0
5
10
15
20
25
30
35
40
45
50

Supplementary file 3

Relative content of total phenolics and groups of phenolic compounds of M38- and M54-infected grapevine leaves at three time points, in relation to the corresponding controls. Values are ratios and represent mean ± standard deviation of three replicates with three technical replicates each. Different letters indicate a significant difference between different time points for the corresponding samples (ANOVA, Duncan test, $p \leq 0.05$).

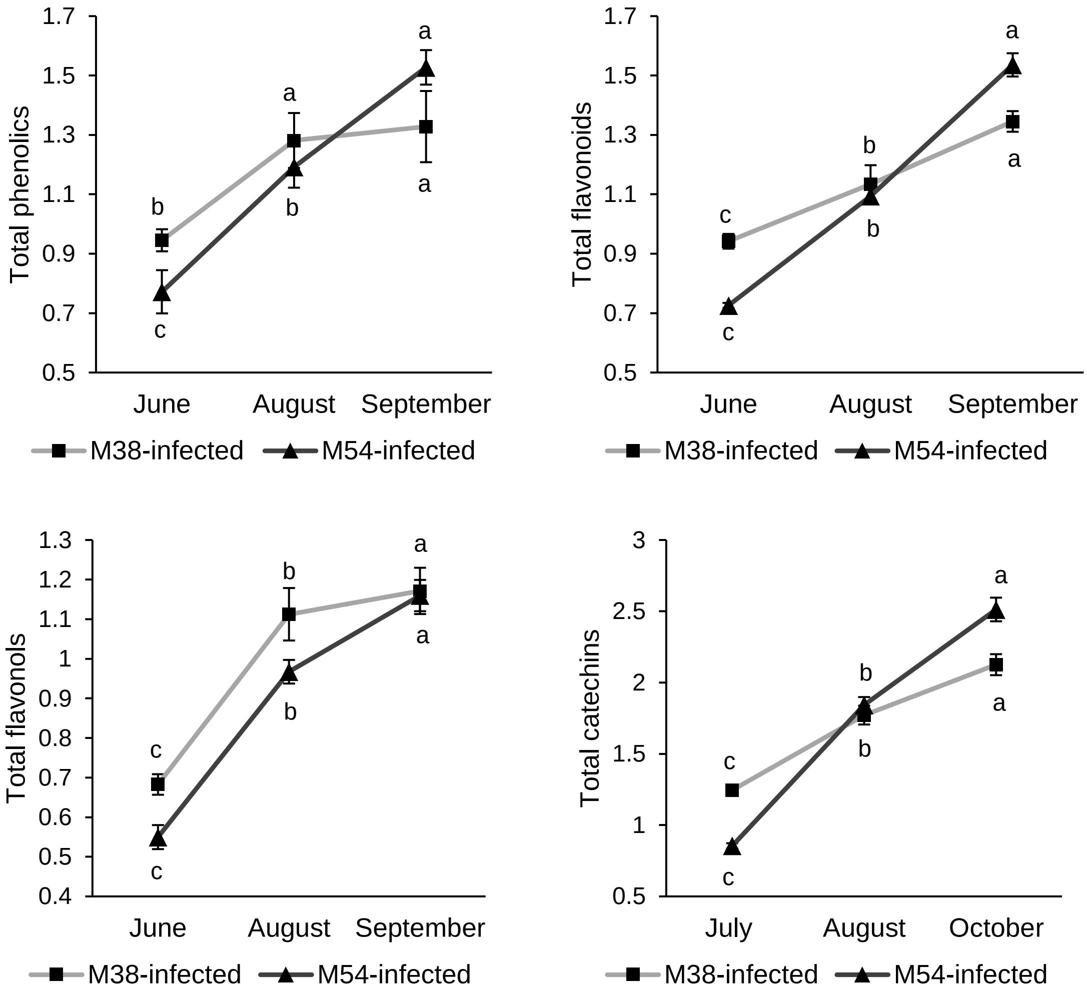

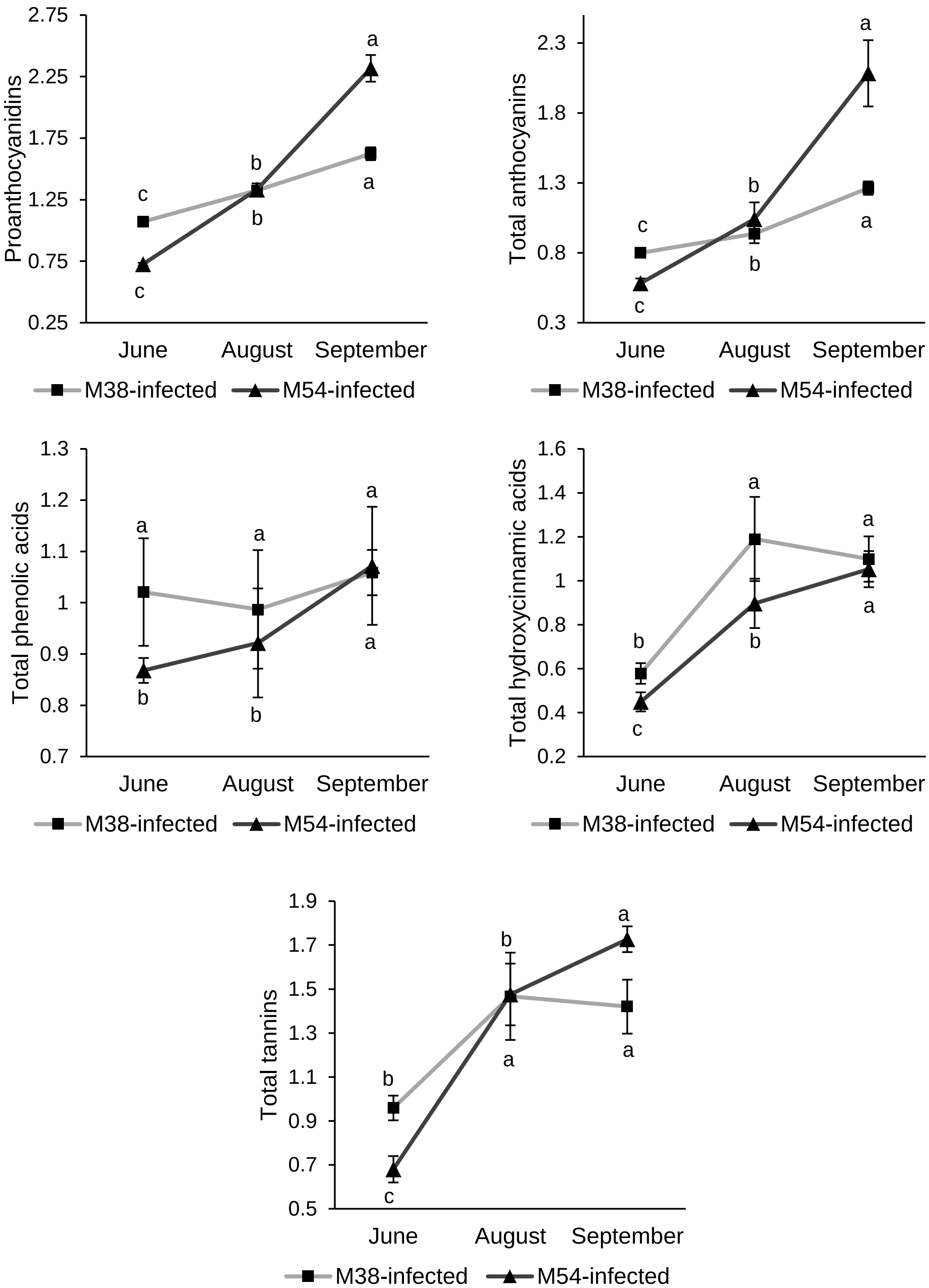
Proanthocyanidins
2.75
2.25
1.75
1.25
0.75
0.25
June
August
September
M38-infected
M54-infected
Total anthocyanins
2.3
1.8
1.3
0.8
0.3
June
August
September
M38-infected
M54-infected
Total phenolic acids
1.3
1.2
1.1
1
0.9
0.8
0.7
June
August
September
M38-infected
M54-infected
Total hydroxycinnamic acids
1.6
1.4
1.2
1
0.8
0.6
0.4
0.2
June
August
September
M38-infected
M54-infected
Total tannins
1.9
1.7
1.5
1.3
1.1
0.9
0.7
0.5
June
August
September
M38-infected
M54-infected

Supplementary file 4

Pearson's correlation coefficients (*r*) between the absolute (A) and relative (B) values of tested parameters. Statistically significant correlations ($p \leq 0.05$) are marked in bold.

A

	ABTS	DPPH	FRAP	TP	TF	TFl	TC	TPAN	TA	TPA	THCA	TT	CA	p-CA	FA	Q	K	Myr	Epicat	Resv	SA	TIP	TIF	TIPA
ABTS	1.00																							
DPPH	**0.86**	1.00																						
FRAP	**0.93**	**0.91**	1.00																					
TP	**0.93**	**0.85**	**0.96**	1.00																				
TF	**0.89**	**0.92**	**0.98**	**0.96**	1.00																			
TFl	**0.72**	0.55	**0.78**	**0.80**	**0.79**	1.00																		
TC	**0.78**	**0.88**	**0.81**	**0.82**	**0.81**	0.35	1.00																	
TPAN	**0.87**	**0.94**	**0.93**	**0.93**	**0.94**	0.57	**0.92**	1.00																
TA	**0.87**	**0.79**	**0.88**	**0.89**	**0.86**	**0.78**	0.61	**0.85**	1.00															
TPA	0.39	0.51	0.61	0.54	**0.69**	0.61	0.33	0.49	0.39	1.00														
THCA	**0.68**	0.43	**0.68**	**0.74**	**0.68**	**0.97**	0.25	0.47	**0.76**	0.45	1.00													
TT	**0.93**	**0.86**	**0.96**	**0.99**	**0.95**	**0.75**	**0.86**	**0.94**	**0.87**	0.49	**0.70**	1.00												
CA	**0.72**	0.44	**0.71**	**0.76**	**0.68**	**0.93**	0.26	0.52	**0.82**	0.45	**0.97**	**0.72**	1.00											
p-CA	0.56	0.25	0.36	0.48	0.32	0.14	0.46	0.43	0.32	0.03	0.22	0.46	0.31	1.00										
FA	**0.86**	**0.75**	**0.89**	**0.97**	**0.92**	**0.83**	**0.71**	**0.86**	**0.89**	0.57	**0.80**	**0.95**	**0.82**	0.49	1.00									
Q	0.46	**0.68**	**0.69**	0.58	**0.75**	0.62	0.50	0.56	0.42	**0.83**	0.43	0.55	0.33	-0.18	0.51	1.00								
K	0.27	0.47	0.41	0.32	0.49	0.22	0.44	0.36	0.02	**0.76**	0.04	0.29	-0.04	0.11	0.27	**0.78**	1.00							
Myr	**0.83**	**0.92**	**0.95**	**0.94**	**0.97**	0.65	**0.88**	**0.98**	**0.82**	0.64	0.53	**0.94**	0.56	0.33	**0.89**	**0.70**	0.46	1.00						
Epicat	**-0.70**	-0.53	-0.50	-0.64	-0.50	-0.44	-0.51	-0.60	**-0.75**	0.17	-0.53	-0.64	-0.56	-0.49	-0.66	0.09	0.31	-0.47	1.00					
Resv	0.67	0.29	0.53	0.62	0.48	**0.80**	0.14	0.36	**0.73**	0.11	**0.90**	0.57	**0.92**	0.39	**0.67**	0.07	-0.26	0.33	**-0.70**	1.00				
SA	0.52	0.41	0.37	0.46	0.34	-0.07	0.67	0.55	0.27	0.03	-0.05	0.48	0.07	**0.86**	0.43	-0.14	0.18	0.45	-0.44	0.06	1.00			
TIP	-0.47	-0.24	-0.21	-0.37	-0.19	-0.17	-0.28	-0.34	-0.53	0.46	-0.32	-0.39	-0.38	-0.47	-0.41	0.43	0.56	-0.18	**0.94**	-0.60	-0.43	1.00		
TIF	0.49	**0.70**	**0.71**	0.60	**0.77**	0.60	0.55	0.60	0.41	**0.85**	0.40	0.57	0.31	-0.10	0.53	**0.99**	**0.83**	**0.72**	0.08	0.05	-0.05	0.42	1.00	
TIPA	**0.80**	0.49	**0.75**	**0.82**	**0.72**	**0.87**	0.39	0.61	**0.83**	0.43	**0.92**	**0.79**	**0.97**	0.53	**0.88**	0.29	0.02	0.62	-0.63	**0.90**	0.29	-0.46	0.29	1.00

B

	ABTS	DPPH	FRAP	TP	TF	TFl	TC	TPAN	TA	TPA	THCA	TT	CA	p-CA	FA	Q	K	Myr	Epicat	Resv	SA	TIP	TIF	TIPA
ABTS	1.00																							
DPPH	**0.99**	1.00																						
FRAP	**0.93**	**0.93**	1.00																					
TP	**0.88**	**0.89**	**0.98**	1.00																				
TF	**0.95**	**0.97**	**0.98**	**0.97**	1.00																			
TFl	0.80	0.81	**0.92**	**0.96**	**0.91**	1.00																		
TC	**0.93**	**0.94**	**1.00**	**0.99**	**0.99**	**0.94**	1.00																	
TPAN	**0.94**	**0.96**	**0.96**	**0.94**	**0.98**	**0.82**	**0.96**	1.00																
TA	**0.93**	**0.94**	**0.91**	**0.88**	**0.93**	0.72	**0.90**	**0.99**	1.00															
TPA	0.72	**0.81**	0.71	0.73	**0.82**	0.67	0.73	0.77	0.72	1.00														
THCA	0.66	0.66	**0.83**	**0.90**	0.81	**0.98**	**0.85**	0.70	0.59	0.59	1.00													
TT	0.81	0.81	**0.97**	**0.97**	**0.91**	**0.94**	**0.96**	**0.88**	0.81	0.62	**0.90**	1.00												
CA	0.79	0.80	**0.96**	**0.97**	**0.91**	**0.94**	**0.96**	**0.87**	0.80	0.62	**0.90**	**1.00**	1.00											
p-CA	-0.64	-0.61	-0.71	-0.63	-0.60	-0.70	-0.70	-0.50	-0.42	-0.30	-0.63	-0.70	-0.69	1.00										
FA	0.80	**0.84**	**0.93**	**0.97**	**0.94**	**0.92**	**0.94**	**0.91**	**0.83**	**0.82**	**0.89**	**0.94**	**0.94**	-0.50	1.00									
Q	**0.92**	**0.93**	**0.93**	**0.94**	**0.96**	**0.95**	**0.95**	**0.88**	**0.81**	0.80	**0.87**	**0.87**	**0.86**	-0.67	**0.90**	1.00								
K	0.53	0.59	0.34	0.37	0.52	0.39	0.39	0.43	0.40	0.81	0.30	0.18	0.17	-0.12	0.42	0.61	1.00							
Myr	**0.90**	**0.92**	**0.98**	**0.98**	**0.98**	**0.90**	**0.98**	**0.98**	**0.94**	0.78	**0.81**	**0.95**	**0.95**	-0.56	**0.97**	**0.91**	0.40	1.00						
Epicat	-0.74	-0.78	**-0.92**	**-0.92**	**-0.88**	**-0.93**	**-0.91**	-0.80	-0.70	-0.72	**-0.89**	**-0.95**	**-0.95**	0.74	**-0.93**	**-0.87**	-0.31	**-0.89**	1.00					
Resv	**0.89**	**0.86**	**0.94**	**0.95**	**0.92**	**0.93**	**0.94**	**0.89**	**0.85**	0.55	**0.87**	**0.92**	**0.91**	-0.65	**0.85**	**0.92**	0.29	**0.91**	-0.80	1.00				
SA	0.79	**0.83**	**0.82**	0.77	**0.82**	0.56	0.80	**0.92**	**0.94**	0.68	0.44	0.74	0.74	-0.31	0.77	0.64	0.25	**0.87**	-0.65	0.67	1.00			
TIP	**-0.90**	**-0.93**	**-0.95**	**-0.91**	**-0.95**	-0.77	**-0.94**	**-0.98**	**-0.96**	-0.78	-0.65	**-0.88**	**-0.88**	0.54	**-0.89**	**-0.83**	-0.37	**-0.97**	**0.83**	**-0.81**	**-0.95**	1.00		
TIF	**0.92**	**0.93**	**0.91**	**0.93**	**0.96**	**0.93**	**0.94**	**0.88**	**0.82**	**0.83**	**0.85**	**0.85**	**0.84**	-0.64	**0.90**	**1.00**	0.65	**0.90**	**-0.85**	**0.89**	0.64	**-0.83**	1.00	
TIPA	0.77	0.79	**0.94**	**0.97**	**0.91**	**0.93**	**0.94**	**0.89**	**0.82**	0.67	**0.90**	**0.98**	**0.99**	-0.57	**0.97**	**0.85**	0.22	**0.96**	**-0.93**	**0.89**	0.76	**-0.88**	**0.84**	1.00

Supplementary file 5

Schematic representation of the main phenylpropanoid pathway with indicated main branches of phenolic metabolism. The content of quantified identified compounds and groups was presented using relative content for M38-infected leaves (upper row) and M54-infected leaves (lower row) for three time points through the development of infection.

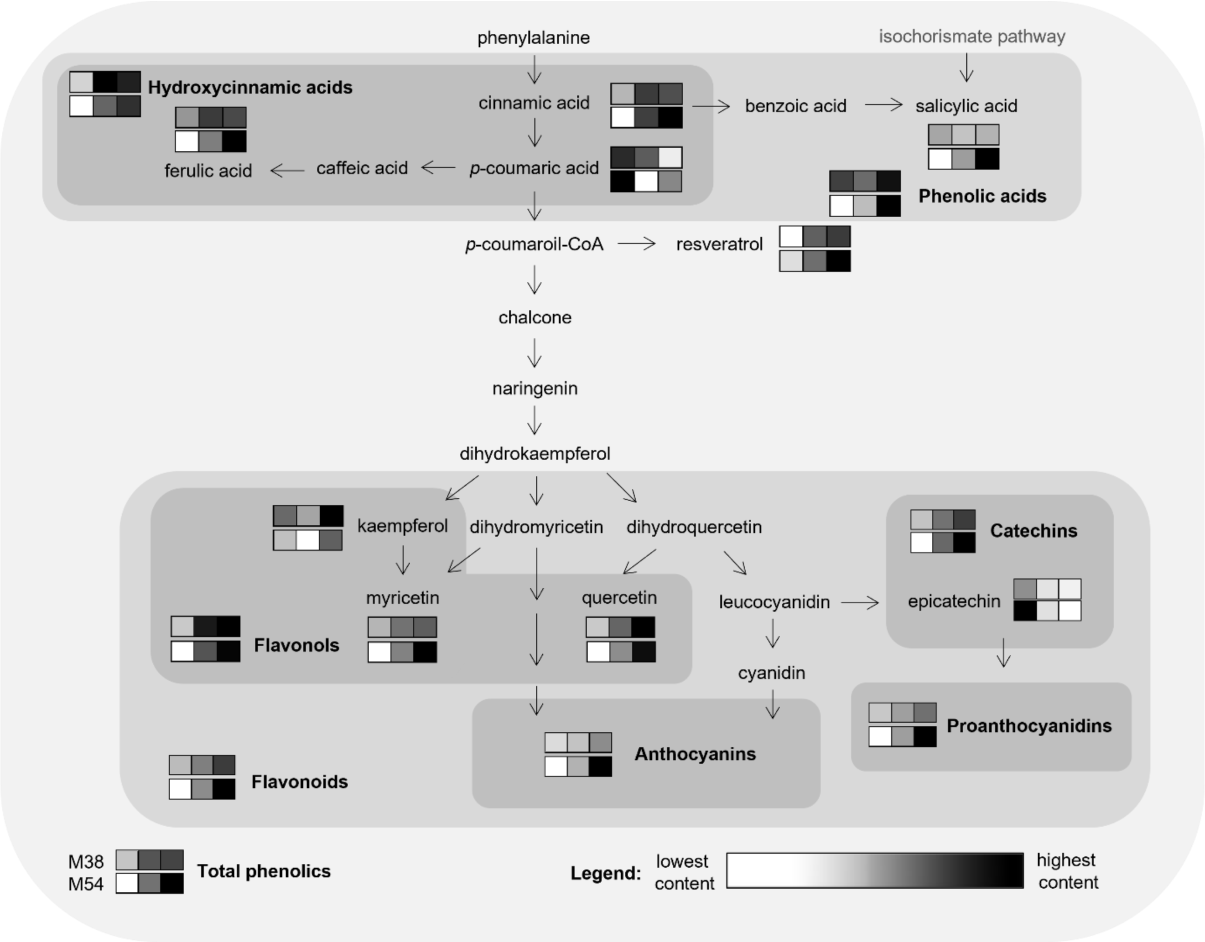

Supplementary file 6

Relative content of individual phenolic compounds of M38- and M54-infected grapevine leaves at three time points, in relation to the corresponding controls. Values are ratios and represent mean ± standard deviation of three replicates. Different letters indicate a significant difference between different time points for the corresponding samples (ANOVA, Duncan test, $p \leq 0.05$).

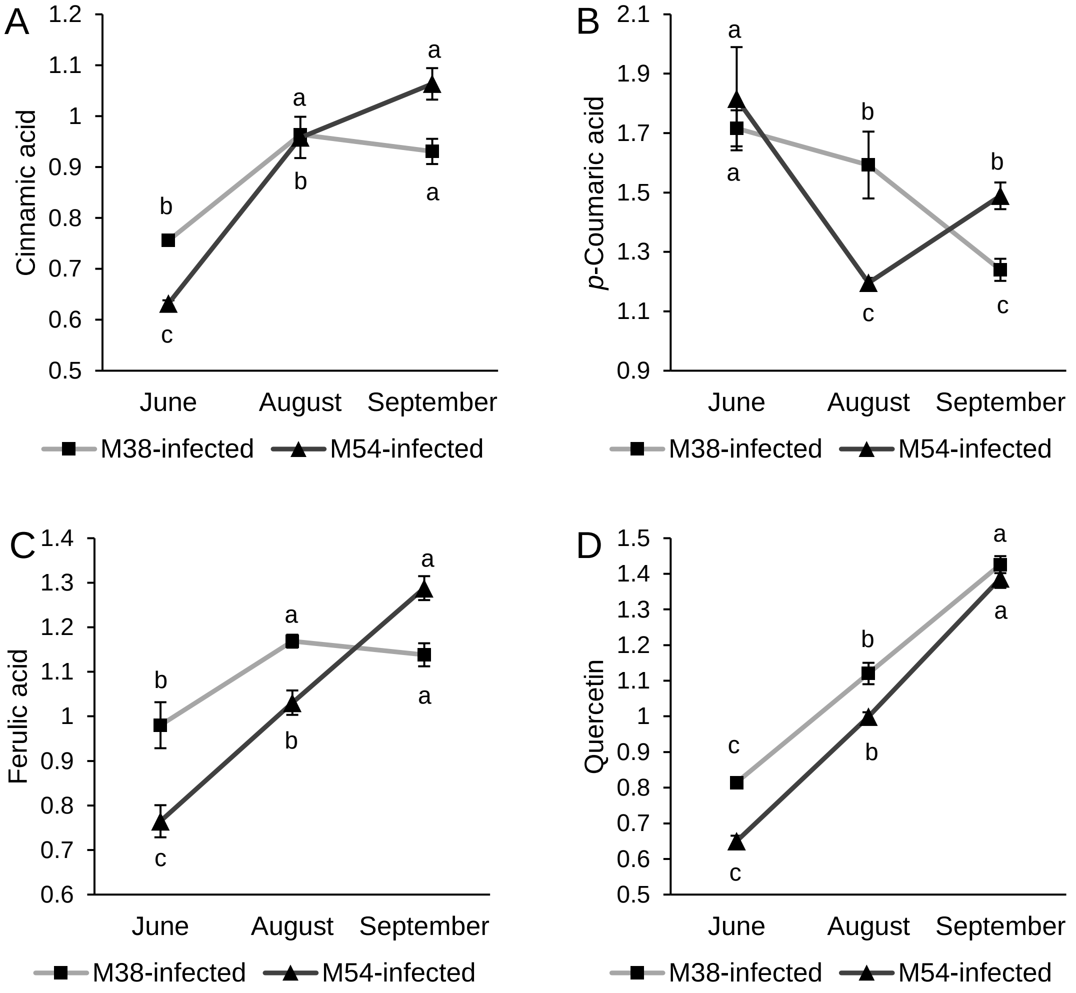

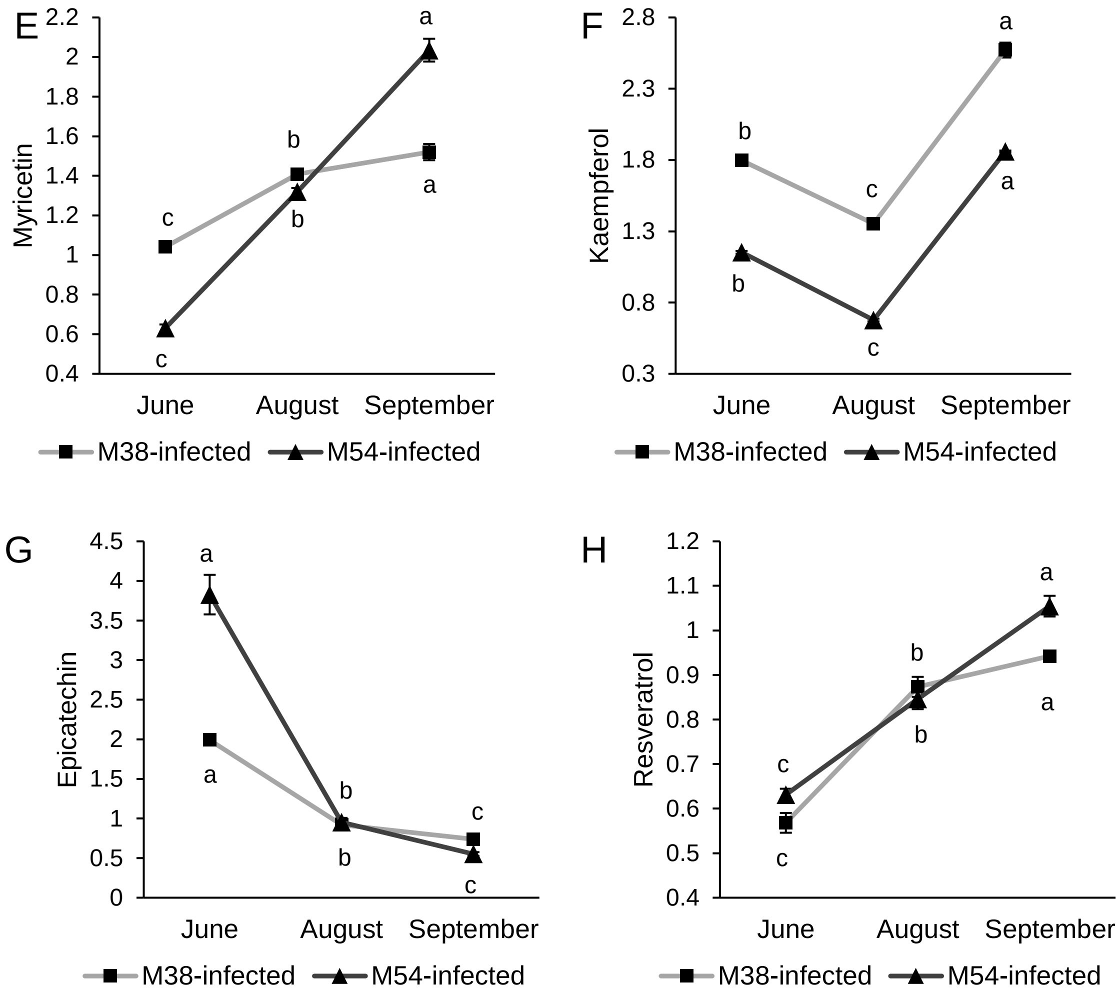
E
Myricetin
2.2
2
1.8
1.6
1.4
1.2
1
0.8
0.6
0.4
June
August
September
M38-infected
M54-infected
F
Kaempferol
2.8
2.3
1.8
1.3
0.8
0.3
June
August
September
M38-infected
M54-infected
G
Epicatechin
4.5
4
3.5
3
2.5
2
1.5
1
0.5
0
June
August
September
M38-infected
M54-infected
H
Resveratrol
1.2
1.1
1
0.9
0.8
0.7
0.6
0.5
0.4
June
August
September
M38-infected
M54-infected

Supplementary file 7

(A) Number of increased, decreased, and resistant (no difference) parameters for M38- and M54-infected leaves in comparison to the corresponding controls for three time points (June, August, September). (B) Number of parameters that had the highest values at each time point (June, August, September) for every analysed group.

A

		↑ increase	↓ decrease	= no difference
Jun	M38-infected	6	9	4
	M54-infected	5	14	0
Aug	M38-infected	13	1	4
	M54-infected	9	2	8
Sep	M38-infected	14	0	4
	M54-infected	14	0	4

B

	M38-infected	M54-infected	uninfected
June	9	2	12
August	16	9	5
September	5	16	5
